# Propolis Stands out as a Multifaceted Natural Product: Meta-Analysis on Its Sources, Bioactivities, Applications, and Future Perspectives

**DOI:** 10.3390/life15050764

**Published:** 2025-05-09

**Authors:** Ahmed Sabri Ayad, Samia Benchaabane, Tarek Daas, Guy Smagghe, Wahida Loucif-Ayad

**Affiliations:** 1 Pharmaceutical Sciences Research Center (CRSP), Constantine 25000, Algeria; sabri.ayad@crsp.dz; 2 Laboratory of Excellence Applied Animal Biology, Faculty of Sciences, Badji Mokhtar University, Annaba 23000, Algeria; benchaabanesamia@yahoo.com (S.B.); tarek63daas@yahoo.fr (T.D.); 3 Department of Plants and Crops, Ghent University, 9000 Ghent, Belgium; 4 Institute of Entomology, Guizhou University, Guiyang 550025, China; 5 Department of Biology, Vrije Universiteit Brussel (VUB), 1050 Brussels, Belgium; 6 Faculty of Medicine, Badji Mokhtar University, Annaba 23000, Algeria

**Keywords:** propolis, sources, chemical composition, biological activities, PRISMA approach

## Abstract

Honeybee (*Apis* spp.) products have been used for centuries due to their nutritional value and diverse healing properties. Propolis, produced by honeybees, is a unique resin collected from tree buds, sap flows, and other plant exudates, which is then mixed with bee enzymes, beewax, and secretions. This comprehensive review starts with a meta-analysis following the PRISMA approach to explore recent advances in the chemical composition of propolis, its biological activities and pharmacological properties, its applications and products, and future perspectives. The composition of propolis varies depending on plant source, season of harvest, geography, type of bee flora, climate, and honeybee species at the site of collection, and some of these are related. Flavonoids, aromatic acids, phenolic acids, and their esters are key bioactive compounds in propolis, contributing to their diverse pharmacological properties, such as antioxidant, antibacterial, antiparasitic, antiviral, antileishmanial, antidiabetic, anti-inflammatory, immunomodulatory, and anticancer effects. In summary, propolis stands out as a multifaceted natural product with a broad spectrum of biological activities. This review aims to provide valuable insights for researchers, practitioners, and decision-makers involved in studying the sources, composition, and biological activities of propolis. The highlighted hotspots and emerging frontiers presented herein are poised to unlock the full potential of propolis, paving the way for innovative applications in health and wellness.

## 1. Introduction

Bee products have been well known and used since ancient times and the Middle Ages for to their nutritional and therapeutic potentials in human health protection [1,2,3,4]. Propolis, one of those bee products, commonly referred to as “bee glue”, is a resinous material produced by honeybees from substances collected from plants, buds, and exudates [1,2]. The term *propolis* originates from ancient Greek, where *pro* means “in front of” and *polis* means “city”, indicating that this natural product is used in hive protection and defense [3,4,5]. Bees use propolis to seal openings and cracks in their hives, smooth internal walls, and protect against external threats such as predators and adverse weather [1,4,6].

Since ancient times, propolis has been identified and used by humans as a traditional and therapeutic medicine [7,8,9,10]. The ancient Egyptians primarily utilized it for embalming their cadavers to inhibit bacterial and fungal proliferation, thus preventing decomposition [4]. They observed this behavior in bees, who embalm intruders they killed but cannot remove from the hive [11]. Greek and Roman physicians employed propolis as an antiseptic, and a mouth disinfectant, and also for treating gastrointestinal diseases (such as stomach ulcers) and promoting wound healing and burn healing [12,13]. Furthermore, British pharmacopeias in the 17th century listed propolis as an official drug [6], and since then it has been widely used as a common natural remedy. During World War II, propolis served as an anti-inflammatory and antibacterial treatment [5].

The chemical composition, characterized by distinct herbal aromatic scents and a range of colors such as brown, green, yellow, and red, depends on factors, including plant origin, geographic location, bee species, seasonality, and type of extraction solutions used [1,14]. Today, with advancements in chemistry, sophisticated analytical techniques allow for the determination of its chemical composition. To date, over 1000 compounds have been identified in propolis, spanning various chemical classes, including phenolic acids, flavonoids, terpenes, and alkaloids [15]. Ongoing research continues to uncover new bioactive molecules within propolis [16,17], and further elucidate its biological properties, including antioxidant, antibacterial, fungicidal, antidiabetic, anticancer, anti-inflammatory, and immunomodulatory activities [18,19,20,21,22,23,24].

Currently, propolis is widely utilized in various cosmetic and healthcare products, such as gels, creams, throat sprays, cough syrups, and mouthwashes [25,26]. The aim of this review is to summarize and discuss the chemical composition and biological activities of propolis, while exploring its possible applications and future perspectives as a multifaceted natural product.

## 2. Methodology

A comprehensive literature search was conducted using PubMed (https://pubmed.ncbi.nlm.nih.gov/, accessed on 15 February 2025) [27] to gather relevant studies published over a period of 25 years, from January 2000 to February 2025. A variety of keywords were employed to maximize the retrieval of pertinent articles, focusing on ‘‘propolis’’ AND ‘‘propolis extraction’’ AND ‘‘composition of propolis’’ AND ‘‘biological activities of propolis’’ AND ‘‘application of propolis’’ AND ‘‘formulation of propolis’’ (Table 1). A total of 11,047 articles were retrieved (Table 1).

**Figure 1:**
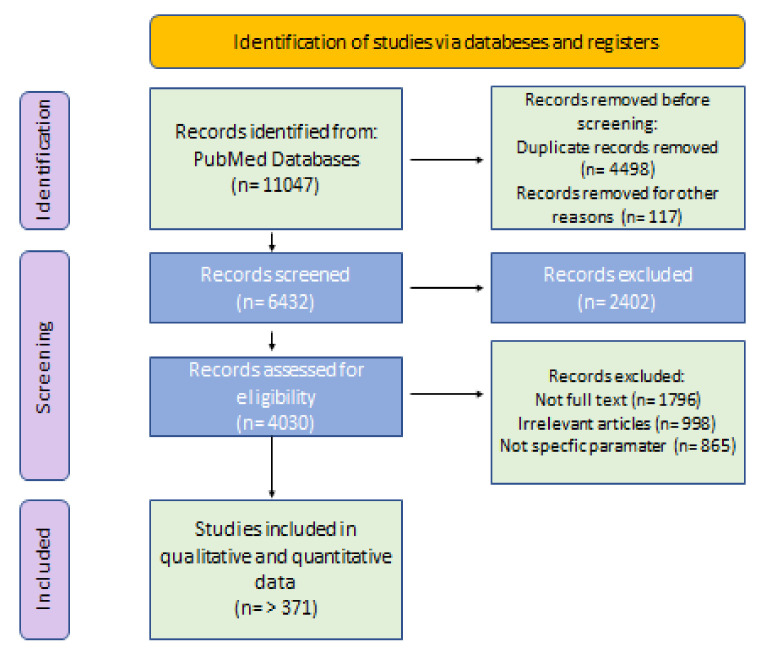
PRISMA flowchart method for literature search on propolis in PubMed database (https://pubmed.ncbi.nlm.nih.gov/, accessed on 15 February 2025) from January 2000 to February 2025.

As shown in Figure 1, the PRISMA flow diagram for systematic reviews was employed to guide the processes of screening, eligibility assessment, and inclusion. The PRISMA methodology was applied to identify relevant articles and eliminate records before screening, with over 4000 entries initially retrieved. During the screening phase, records were reviewed, relevant reports were identified and gathered, eligibility was assessed through exclusion criteria, and finally, 371 reports were selected for inclusion in the study.

**Figure 2:**
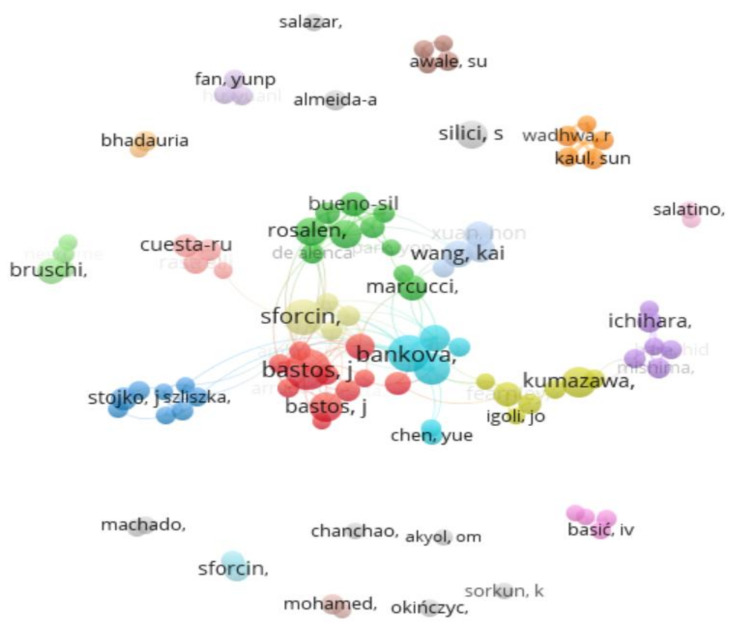
Authors and co-authors involved in the research of propolis, based on a comprehensive literature search in PubMed database (https://pubmed.ncbi.nlm.nih.gov/, accessed on 15 February 2025) from January 2000 to February 2025, as presented using VOSviewer.

The VOSviewer program (https://www.vosviewer.com/, accessed on 15 February 2025) [28] was utilized to create a network of authors and co-authors (Figure 2). A bibliometric analysis was performed to identify key contributors to propolis research and related fields. By mapping author collaborations, the study aimed to reveal patterns of cooperation and highlight influential researchers. The author network visualization in Figure 2, generated using VOSviewer, was based on a predefined threshold—only authors with a minimum of 10 published documents were included.

Among the 20,521 authors analyzed, 97 met the inclusion threshold, leading to the identification of nine distinct clusters based on co-authorship connections. The size of each author’s representation, depicted as a circle, corresponded to their number of publications, facilitating the visualization of key contributors in the field. Authors with the highest publication counts were represented by circles with larger diameters, reflecting their significant contributions. Bastos Jairo Kenupp, Sforcin José Mauricio, Bankova Vassya, Rosaken Pedro Luiz, Popova Milena, and Berretta Andresa Aparecida exhibited the highest recorded documents on VOSviewer, with 101, 76, 66, 51, 41, and 41, respectively.

Table 2 highlights some of the most prominent institutions recognized for their contributions to propolis research between 2000 and 2025. These institutions, among others; are known for their studies on the chemical composition, biological properties, and applications of propolis.

**Figure 3:**
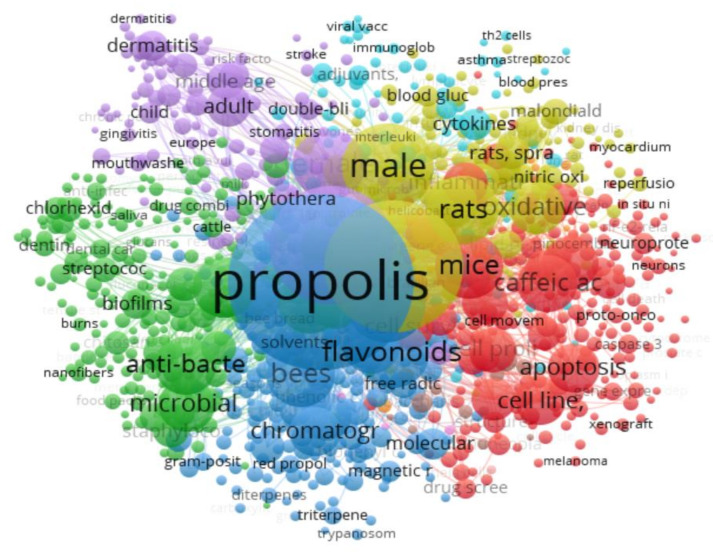
Co-clustering map of high-frequency keywords reported in papers about propolis, following the comprehensive literature search in the PubMed database (https://pubmed.ncbi.nlm.nih.gov/, accessed on 15 February 2025) from January 2000 to February 2025, and presented using VOSviewer.

VOSviewer (https://www.vosviewer.com/program, accessed on 15 February 2025) [28] was utilized to examine keyword co-occurrences. Data retrieved from PubMed were exported as a RIS file, uploaded to VOSviewer, and analyzed using the “Create a map based on bibliographic data” function. A threshold of at least five occurrences was applied, selecting keywords that met this criterion. Among the 10,739 original keywords, 1566 qualified, and a final selection of 1000 keywords was made. The co-occurrence network identified five primary keyword clusters related to propolis, with the most prominent keywords being *propolis*, *animals*, *humans*, *antioxidants*, and *flavonoids* with respective values of 3338, 1863, 1508, 614, and 518 (Figure 3).

**Figure 4:**
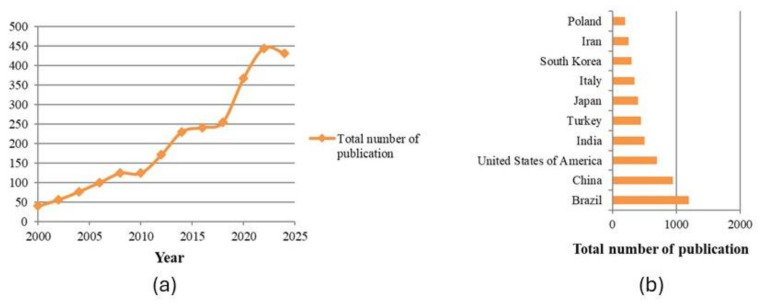
Research trends on propolis based on the comprehensive literature search in the PubMed database (https://pubmed.ncbi.nlm.nih.gov/, accessed on 15 February 2025) from January 2000 to February 2025: (**a**) number of publications on propolis; (**b**) country-based research trends showing the percentage of publications on propolis.

Over the past 25 years, research on propolis has gained increasing attention, particularly following the COVID-19 pandemic (Figure 4a), which highlighted the importance of natural compounds with potential antiviral, antibacterial and immune-modulatory properties. Scientists and researchers worldwide have intensified their efforts to explore the diverse chemical composition, biological activities and potential therapeutic applications of propolis. Notably, countries such as Brazil, China, the United States of America, India, Turkey, Japan, Italy, South Korea, Iran, and Poland have emerged as leading contributors to scientific advancements in this field (Figure 4b). Their research efforts span various disciplines, including pharmacology, microbiology, and biotechnology, aiming to better understand the potential of propolis in modern medicine, functional foods, and pharmaceutical applications.

## 3. Composition of Propolis and Extractive Procedures

**Figure 5:**
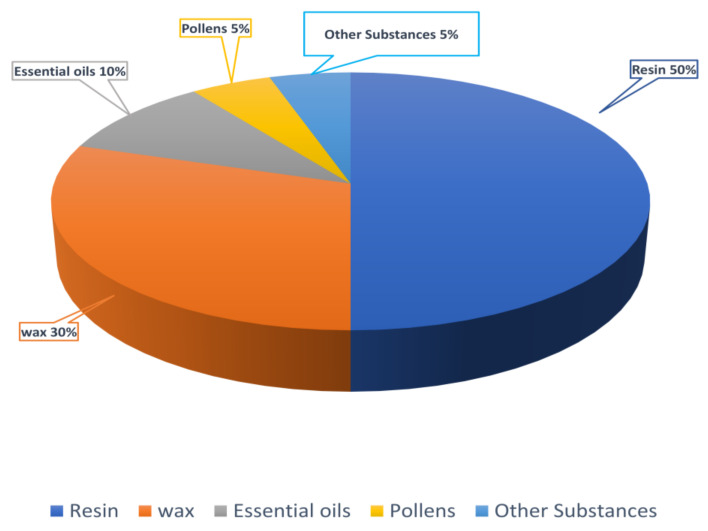
Gross chemical components of propolis.

The chemical profile of propolis differs based on the geographical region where bees collect their raw materials and the available flora, but it typically contains resins, beeswax, essential oils, and pollen (Figure 5). Propolis has been used in traditional medicine for its potential antibacterial, antiviral, antifungal, and anti-inflammatory activities. Historically, it has been applied to wounds, used to treat abscesses, and employed in various remedies [4].

Based on geographic origin and plant source, researchers have categorized propolis into seven major types [29,30]:

- Poplar (China, New Zealand, Europe, and America) originates mainly from the bud exudates of *Populus species* [31]. It is rich in flavonoids such as chrysin, galangin, and pinocembrin.
- Birch (Russia) originates from *Betula verrucosa* Ehrh, and is characterized by high levels of phenolic acids and their esters [5].
- Mediterranean (Malta, Sicily, Crete, and Greece) is mainly collected from the resin of *Cupressus sempervirens*, and is noted for its abundance of diterpenes [6].
- Green (Southeastern Brazil) is derived from *Baccharis dracunculifolia* [32], distinguished by its high content of prenylated phenolic compounds such as artepillin C.
- Red (Southeast Mexico, Northeastern Brazil, and Cuba) is collected from resins of *Dalbergia ecastaphyllum* [33], and is rich in isoflavonoids and polyphenolic compounds.
- Brown (some regions of Brazil, Venezuela, and Cuba) is collected from the resins of *Clusia* species and *Copaifera* species [34], characterized by the presence of benzophenones and polyisoprenylated benzophenones.
- Pacific (Hawaii, Taiwan, and Indonesia) is collected from *Macaranga* species and contains significant amounts of prenylated stilbenes and other phenolic compounds [4,35,36].

Across all types, the major chemical groups present in propolis include flavonoids, phenolic acids or their esters, terpenes, aliphatic and aromatic acids, fatty acids, alcohols, β-steroids, and alkaloids [37,38].

Flavonoids such as flavones (luteolin), flavonols (quercetins and derivatives), flavanones (pinocembrin, 5,7-dihydroxyflavone and derivatives, naringenin), flavanonols (garbanzol and alnustinol), chalcones and dihydrochalcones, isoflavones (calycosin), isodihydroflavones (daidzein), flavans, isoflavans (vestitol and derivatives), and neoflavonoids (homopterocarpin and medicarpin) are the primary compounds responsible for the pharmacological properties of propolis [39]. Phenolic compounds, including cinnamic acid, pinocembrin, p-coumaric, chrysin, caffeic and fulvic acids, naringenin, galangin, caffeic acid phenylethyl ester (CAPE), apigenin, and quercetin, also contribute to its biological properties [4,25].

Although terpenoids constitute only about 10% of the composition, they are responsible for the characteristic odor of propolis and serve as key factors in differentiating various types. These include monoterpenes (terpineol, camphor), diterpenes (the main group, such as ferruginol, junicedric acid and derivatives, pimaric acid, and totarolone), triterpenes (such as lupeol and derivatives, lanosterol, and amyrone and derivatives), and sesquiterpenes (such as β-elemene, valencene, α-ylangene, and α-bisabolol) [38].

Propolis has also proved to be a rich source of minerals (Ca, Mg, I, K, Zn, Cu, Mn, Fe, and Na) and vitamins (B1, B2, B6, C, and E), which are fundamental for various structures and functions [40]. Furthermore, enzymes such as glucose-6-phosphate, adenosine triphosphate, acid phosphate, and succinic dehydrogenase have been identified in various types of propolis [38,41,42].

Moreover, amides, proteins, amines, and amino acids (glutamic acid, methionine, aspartic acid, tryptophan, phenylalanine, leucine, cysteine, lysine, serine, histidine, arginine, proline, tyrosine, alanine, threonine, and valine) have been found in propolis, serving as sources of nitrogen [38].

Indeed, through various high throughput chromatographic technics, such as high-performance liquid chromatography (HPLC), gas chromatography–mass spectrometry (GC-MS), thin-layer chromatography (TLC), and nuclear magnetic resonance (NMR) [43,44], over 1000 different molecules from various origins have been identified [15,45]. It should be noted here that the chemical profile of propolis extracts is influenced by the choice of solvent [15]. Numerous studies have employed solvents such as ethanol, hexane, water, ethyl ether, chloroform, methanol, seed oil, and acetone. It was concluded that ethanol, particularly at a concentration of 70–75%, is the most suitable and effective solvent for extracting propolis [25,46].

Many studies have reported variations in the chemical composition and biological properties of propolis extracts based on the extraction technique used, emphasizing the influence of the method on the yield and selectivity of certain compounds [47,48,49,50], and consequently on the biotechnological potential of the extracts.

Due to limitations associated with conventional liquid propolis extracts, newer, more environmentally friendly extraction methods have been developed, including maceration, Soxhlet extraction (SE), ultrasonic-assisted extraction (UAE), microwave-assisted extraction (MAE), and supercritical CO_2_ extraction. These newer methods are designed to achieve higher yields while consuming less energy and time, making them more economically efficient for the industrial applications, particularly in the production of functional foods [51,52].

Pellati et al. [53] described the use of UAE and MAE of propolis extraction using an ethanol–water mixture of (80:20, *v*/*v*) and compared their effectiveness with traditional maceration and conventional heat reflux extraction (HRE). Their findings demonstrated that modern technologies achieved extraction efficiencies comparable to traditional methods while operating within similar or shorter processing times.

Furthermore, many studies have shown that UAE can efficiently enhance the extraction process, allowing for the rapid recovery of a wide range of compounds while minimizing the use of organic solvents and operating at lower temperatures [15]. This approach reduces energy consumption and it is crucial for preventing the thermal degradation of sensitive active compounds [48,54,55,56].

Sambou et al. [57] compared SE, MAE, and UAE using ten extraction solvents for their efficiency in extracting phenolic and flavonoid compounds, antioxidants, and 5-lipoxygenase inhibitory compounds from eastern Canadian propolis. Their results showed that UAE was effective in achieving higher yields in shorter times with preserving the high biological activity of the extracts.

Reis et al. [58] and Khacha-Ananda et al. [59] reported that extracts obtained by UAE had higher concentrations of phenolics and flavonoids and exhibited greater antimicrobial, antioxidant, and cytotoxic activities. These results were confirmed by Ding et al. [60], who demonstrated that UAE significantly increased the yield of total phenolics and flavonoids, thus enhancing the antimicrobial activity of the extracts.

The enhancement achieved through UAE generally attributed to various mechanical effects, such as the disruption of cellular structures, which increases mass transfer and the contact surface area between the solvent and the material [55,61,62]. Consequently, the acceleration of the extraction process is remarkable. Oliver et al. [63] used microscopy to illustrate the effects of ultrasound on wax, showing that the process led to a redistribution of the wax within the sample, thereby improving substrate accessibility. By fragmenting and dispersing particles, UAE facilitated the incorporation of waxy propolis material into the extract, eliminating the need for an overnight step.

## 4. Biological Activities of Propolis

### 4.1. Antioxidant Activity

Oxidative stress is recognized as a major public health problem. It results from an imbalance between free radicals and antioxidants in the body, leading to cell damage and contributing to various any diseases, such as heart disease, atherosclerosis, and cancer [64]. To prevent oxidative stress and avoid the side-effects of synthetic antioxidant drugs, natural products have emerged as valuable sources of antioxidant compounds, with propolis being recognized as one of the richest in bioactive components [65].

Numerous studies have demonstrated the antioxidant activity of propolis using the DPPH assay, which is based on the reduction in the 2,2-diphenyl-1-picryl-hydrazyl-hydrate (DPPH•) radical in the presence of an antioxidant, resulting in the formation of the non-radical DPPH-H form at the end of the reaction [66]. The DPPH assay, which is the most frequently used method and relies on a color change in the solution, is a valid, easy, accurate, sensitive, and cost-effective method to evaluate scavenging activity of antioxidants in extracts. Since the DPPH radical is stable, it does not need to be generated (via chemical reactions or external factors), unlike other scavenging assays [65,66].

It should be noted that antioxidant activity should not be determined using a single test model; instead, employing multiple evaluation methods is recommended for a more comprehensive assessment [65,67,68]. Several studies worldwide have investigated different propolis samples using of other assays, such as those based on 2,2′-azino-bis(3-ethylbenzothiazoline-6-sulfonic acid) (ABTS) scavenging activity, ferric reducing antioxidant power (FRAP), and β-carotene bleaching [60,69,70,71,72,73]. These methods have relatively simple and rapid protocols. However, they are based on different mechanisms, with different mechanisms, with distinct reaction environments, principles, and charged radicals, and utilize different reference compounds. As a result, comparing data obtained from these methods can be challenging [45].

Flavonoids and phenolic acids are believed to be primarily responsible for the antioxidant activity of propolis [36,74,75,76,77]. The flavonoids in propolis are powerful antioxidants, capable of scavenging free radicals by interacting with the reactive components of the radical, thereby rendering them inactive [78]. These compounds protect cells from damage [79] and lipid peroxidation [80,81]. Additionally, the phenolic compounds in propolis and their esters block and suppress the generation of reactive oxygen species (ROS) [20,80]. This antioxidant activity in propolis has been attributed to several of its components, such as quercetin, pinocembrin, CAPE, kaempferol, artepillin-C, caffeic acid, galangin, benzyl caffeate, pinocembrin, ferulic acid, p-coumaric acid, flavones, and flavanols [73,82,83,84]. However, it is challenging to identify the most effective components, as several bioactive compounds in propolis may act synergistically to enhance antioxidant activity [85,86].

It is important to note that propolis from different geographical locations exhibits varying antioxidant activity, despite differences in chemical composition [36,87]. This variability is due to factors such as plant origin, geographic location, bee species, temperature variations, seasonality, and storage conditions [69,70,88,89]. Furthermore, the method of extraction and the type of solvent used can influence the chemical composition of propolis extracts [90,91]. These factors contribute to the variability in antioxidant activities observed in different propolis extracts worldwide, making comparisons between results difficult or, in some cases, not feasible.

In vivo studies, particularly using mouse models such as C57BL/6 mice with acute lung inflammation caused by cigarette smoke, have shown that propolis extract enhances the activity of superoxide dismutase, glutathione peroxidase, and catalase, while reducing malondialdehyde levels and decreasing the glutathione/oxidized glutathione ratio [92]. Da Silveira et al. [93] demonstrated a decrease in the production of nitric oxide and malondialdehyde in 3-month-old male Wistar rats, without affecting the levels of antioxidant enzymes superoxide dismutase and catalase. Sun et al. [94] showed that propolis strengthens the body’s antioxidant defense system, preserves vitamins, and reduces hydroperoxide activity. Remirez et al. [95] and Lima et al. [96] demonstrated a protective effect of propolis against liver injury and DNA damage in rats induced by allyl alcohol and 1,2-dimethylhydrazine, respectively. The bioactive compounds in propolis exhibit strong antioxidant properties, effectively neutralizing free radicals and protecting cells from various forms of macromolecular damage.

### 4.2. Antimicrobial Activity

Microorganisms, including bacteria, viruses, fungi, and others, are responsible for infectious diseases in humans and exhibit significant genome instability along with a strong ability to adapt to changing environmental conditions. Due to the increasing failure of synthetic microbial drugs, there is growing interest in natural products like propolis as sources of new bioactive molecules [7].

#### 4.2.1. Antibacterial Activity

The antibacterial properties of propolis have been recognized since ancient times. Bees utilize propolis to maintain a sterile environment within their hives, acting as a natural disinfectant [3]. This characteristic has sparked scientific interest in exploring the antibacterial potential of propolis collected from various regions worldwide. The ethanolic extract of propolis is the most commonly used form, primarily because of its effectiveness in extracting the bioactive compounds present. Table 3 summarizes the susceptibility of several bacterial strains, both Gram-positive and Gram-negative, to propolis extracts.

**Table 3:** Activity of propolis extracts (using different solvents) from various geographical regions (country/region), listing their major components and antibacterial effects different Gram-positive and Gram-negative bacteria, as tested in various methods.

Country/Region	Solvent	Major Components	Method	Bacteria Tested	Key Results	Ref.
Poland	Ethanol or propylene glycol	/	Agar well diffusion.	*E. coli*, *S. aureus*	Propolis extracts in ethanol or propylene glycol showed antibacterial activity against *S. aureus*. Both extracts exhibited similar activity against *S. aureus*.	[12]
China	Ethanol	flavonoids as galangin, pinocembrin, and pinobanksin.	Disk diffusion	*Bacillus subtilis*, *Escherichia coli*, *Staphylococcus aureus*, *Listeria monocytogenes*	All the extracts showed high antimicrobial activity against *S. aureus*, *L. monocytogenes* and *B. subtilis*, but no effect on *E. coli*	[60]
Brazil	Ethanol	The flavonoids and aromatic compounds.	Microdilution	*E. coli*, *S. aureus*	The extracts demonstrated activity against *S. aureus* and *E. coli*, while the activity was higher against *S. aureus*	[84]
Algeria	Ethanol	Phenolics and flavonoids.	Disk diffusion	*S. aureus*, *Bacillus cereus*, *E. coli*, *Pseudomonas aeruginosa*	Ethanolic extract of Algerian propolis samples inhibited growth of all examined microorganisms with the highest activity against Gram-positive bacteria	[97]
Romania	Water	/	Well diffusion. Microdilution	*P. aeruginosa*, *E. coli*, *S. aureus*, *B. cereus*	Inhibitory zones of samples varied from 7 to 17 mm compared to ciprofloxacin with inhibitory zones between 25 and 30 mm. Synergistic interaction between propolis and honey	[98]
India	Ethanol	/	Microdilution	Multidrug-resistant bacteria, *Acinetobacter* sp., *Enterobacter* sp., *Strenotrophomonas* sp.,	Twenty antibiotics showed low inhibitory zones due to resistance. Combination of propolis extract with phenethyl caffeate, chrysin and galangin enhanced the antibacterial activity	[99]
Morocco	Ethanol	Flavonoids enriched	Agar well diffusion. Microdilution	Gram-positive and Gram-negative	Gram-positive bacteria were more sensitive than Gram-negative. Variation in activity depends on the propolis origin. Inhibitory zones of propolis varied from 10 to 22.5 mm compared to chloramphenicol with inhibitory zones between 19 and 37 mm. MIC values ranged from 0.15 to 5 mg/mL, and MBC values varied from 1.25 to 5 mg/mL. Chloramphenicol showed MIC and MBC values between 0.0002 and 0.064 mg/mL	[100]
Europe	Ethanol or water	Phenolic acid esters and Flavonoids	Microdilution. Checkerboard. Dilution and time-kill curve assays	32 reference strains (Gram-positive, Gram-negative and fungi). One strain of methicillin-resistant *S. aureus* (MRSA). One strain of vancomycin-resistant *enterococci* (VRE)	All samples showed moderate activity against Gram-positive. Ethanol-based propolis extracts generally demonstrated moderate effectiveness against Gram-negative bacteria, whereas aqueous extracts exhibited lower activity against these microorganisms. The propolis extract synergistically enhanced the efficacy of antibiotics	[101]
Turkey	Ethanol	Higher phenolics and flavonoids contents.	Agar well diffusion. Microdilution	*S. aureus*, *E. coli*, *P. aeruginosa.*	Propolis extract was more potent in inhibiting *S. aureus* than *E. coli.* Propolis extracts showed no activity against *P. aeruginosa*	[102]
Iran	Ethanol	/	Agar dilution	*S. aureus*, *Streptococcus mutans*, *Lactobacillus acidophilus*, *Enterococcus faecalis*	The lowest MIC values were scored for *S. aureus*, while the highest for *L. acidophilus*	[103]
Egypt, China, Bulgaria, Span, Australia, Greece, Italy, Canada	Ethanol	Various phenolics and flavonoids	Microdilution	*S. aureus*, *E. coli*	All propolis samples showed an inhibition in the growth of all examined microorganisms, but the inhibition varied depending on propolis origin. Propolis from Canada and Egypt showed the highest activity against *S. aureus*, while the propolis from Spain, Greece and Egypt was strongest against *E. coli*	[104]

Many methods, as recommended by the National Committee for Clinical Laboratory Standards, have been employed to evaluate the antibacterial activity of propolis [105]. The agar diffusion method is often used for screening samples against a variety of microorganisms, with results expressed as the diameter of the inhibition zone where bacterial growth is absent. Microdilution and macrodilution techniques provide quantitative data, specifically the minimum inhibitory concentration (MIC) and the minimum bactericidal concentration (MBC), respectively. However, it should be noted that comparing results from studies remains difficult due to variations in the composition of propolis and the methods used for evaluation [105]. The variability in microbiological results between different propolis samples/extracts can be explained by factors such as geographical origin, plant diversity, water content, climate, and bee species, all which influence the formulation and characteristics of propolis [18,60,106,107].

According to the literature, propolis from Algeria [97], Kenya [108], Egypt, China, Bulgaria, Spain, Australia, Greece, Italy [74], Brazil [109], Portugal [110], Iran, and many other countries [98,99,100] exhibited a stronger activity against Gram-positive bacteria, with limited or no effect against Gram-negative bacteria. The greater sensitivity of Gram-positive bacteria to propolis extracts has been attributed to structural differences between bacterial groups [101]. Gram-negative bacteria possess an outer membrane with a higher lipid and polysaccharide content, which can prevent the entry of bioactive molecules into the cell membrane [60,102]. Moreover, Gram-negative bacteria may produce hydrolytic enzymes capable of degrading propolis components [30].

**Figure 6:**
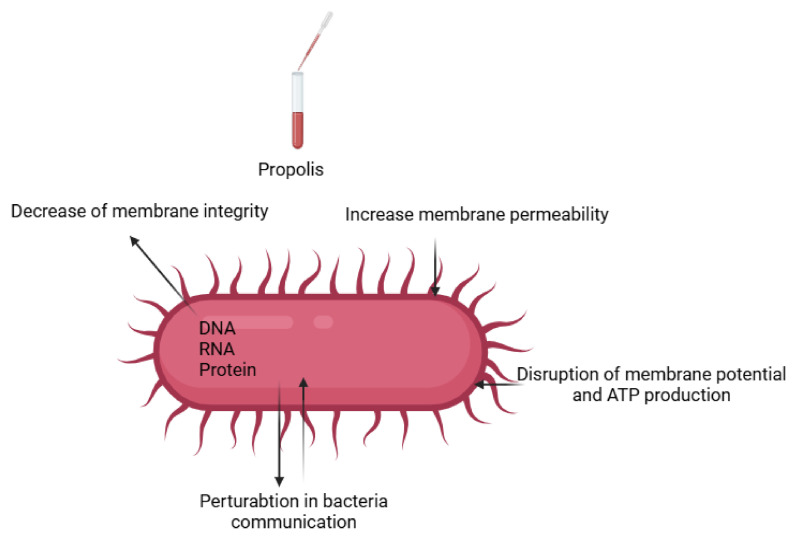
Antibacterial and antiparasitic activities of propolis.

The antibacterial properties of propolis involve various mechanisms critical for bacterial survival and growth, including the inhibition of nucleic acid synthesis [111], disruption of membrane potential, and reduction in adenosine triphosphate (ATP) production [38,112] (Figure 6). Additionally, the type of propolis—and therefore its antibacterial compounds—varies significantly depending on plant sources and geographical region. For instance, Turkish propolis is mainly derived from poplar bud exudate [113,114], containing pentenyl and aromatic caffeates, pinocembrin, pinobanksin-3-O-acetate, and galangin, which serve as taxonomic markers for poplar propolis [42]. Meanwhile, European propolis is characterized by flavonoids and cinnamic acid derivatives, and Brazilian propolis by diterpenic acids and prenylated coumaric acids [42].

The bactericidal action of propolis is largely linked to its higher concentration of phenolic and flavonoid compounds, especially CAPE, galangin, cinnamic acid, pinocembrin, pinobanksin, and ferulic acid [12,60,115]. However, due to the complex composition of propolis, which includes over 1000 compounds, it is difficult to attribute activity to a single molecule. This suggests that its antimicrobial activity results from a synergistic effect among its various components [84].

Moreover, several researchers [116,117] have tested pure compounds identified in propolis and demonstrated that caffeic acid, cinnamic acid, coumaric acid, quercetin, and ferulic acid exhibited antibacterial activity. Nonetheless, the antibacterial activity of natural propolis is higher compared than that of individual compounds, further supporting the idea of a synergistic interaction [100].

The growing phenomenon of bacterial resistance to synthetic antibiotics has stimulated interest in natural products such as propolis for combating multidrug-resistant (MDR) bacterial strains. Several studies have reported the bactericidal effects of propolis samples against clinically isolated MDR bacteria. For example, Stepanović et al. [118] demonstrated that Serbian propolis exhibited strong antibacterial activity against MDR Gram-positive and Gram-negative clinical isolates, with inhibition zones ranging from 6 to 13 mm and 1 to 6 mm (excluding the hole size), respectively. Astani et al. [119] revealed that propolis had both bacteriostatic and bactericidal activity against MDR *Staphylococcus aureus* (MRSA), and vancomycin-resistant *Enterococcus faecium* (VRE). Boufadi et al. [116] reported that Algerian propolis samples increased inhibition zone diameters in a dose-dependent manner against four pathogenic bacterial strains, although strong resistance was observed against nonpathogenic strains of *Shigella dysenteriae* (CECT 524) and *Shigella sonnei* (CECT 584). Propolis from Bulgaria has shown effectiveness against a wide range of anaerobic bacteria, including species of *Propionibacterium*, *Bacteroides*, and *Clostridium* [120]. Additionally, oblongifolin and xanthochymol, compounds identified in propolis, have demonstrated antimicrobial activity against MDR bacteria [121]. The diverse mechanisms of propolis have also led researchers to explore its use in combination with antibiotics as a strategy to combat microbial drug resistance.

Certain studies have reported that combining propolis extracts (at 10% concentration) with topical antibiotics enhanced antibacterial activity against *S. aureus* strains isolated from abscesses and infected wounds, with the most significant effects observed when combined with bacitracin, gentamicin, and oxytetracycline [122]. Furthermore, the synergistic action of Irish propolis in combination with oxacillin and vancomycin against MRSA strains has been confirmed. The combination of propolis and levofloxacin also demonstrated increased efficacy against *Haemophilus influenzae* and *Streptococcus pneumoniae*, pathogens associated with respiratory infections [101].

The synergistic effect of propolis ethanolic extract with ciprofloxacin against clinically relevant *P. aeruginosa* and *S. aureus* has also been described [119,123]. These studies showed that propolis extracts enhanced the antibacterial effect of antibiotics during pathogenesis. Other studies have reported synergistic interactions between propolis and antibiotics such as gentamicin, chloramphenicol, tobramycin, netilmicin, linezolid, and tetracycline, demonstrating efficacy against both drug-sensitive and drug-resistant bacterial strains [124,125].

In vivo studies have shown that administration of ethanolic propolis extract at 300 mg/kg body weight for 30 days provided strong antibacterial effects against *Salmonella* in infected BALB/c mice [126]. Similarly, Santiago et al. [127], Bazvand et al. [128], Mohan et al. [109], and Carbajal Mejia [129] reported that propolis mouthwash has antibacterial properties comparable to those of chlorhexidine (CHX).

Nazeri et al. [103] compared the antibacterial properties of propolis mouthwash with CHX, Listerine, and water in a two-week clinical study using laboratory rats. The results showed that propolis-based mouthwash exhibited superior effectiveness against oral bacteria compared to CHX and Listerine. This finding is consistent with [130] that a 2% propolis mouthwash is more efficient than 0.12% CHX and maintained its effect for up to 45 days. Vasconcelos et al. [131] also showed the positive effect of propolis mouthwash against *E. faecalis*, *S. mutans*, and *S. aureus*.

Given its antibacterial action, propolis could serve as an efficient alternative to support the treatment of diseases, particularly those caused by Gram-positive bacteria, and may also enhance the effectiveness of antibiotic therapy.

#### 4.2.2. Antiparasitic Activity

The antiparasitic activity of propolis from various geographic regions has been evaluated against multiple parasite species, including *Toxoplasma gondii*, *Trypanosoma cruzi*, *Trypanosoma brucei*, *Schistosoma mansoni*, *Trichomonas vaginalis*, *Giardia duodenalis*, *G. lamblia*, and the ectoparasitic honeybee mite, *Varroa destructor*.

*T. gondii* is an intracellular zoonotic protozoan parasite that causes toxoplasmosis, considered one of the most common parasitic zoonoses affecting all vertebrates, including humans and mammals [132]. Barkat et al. [133] and Elmahallawy et al. [134] investigated the potential effects of propolis, and the combination of propolis with wheat germ oil in the treatment of chronic toxoplasmosis in Swiss albino mice using the brain squash technique. These results revealed that propolis reduced and suppressed the multiplication of *T. gondii* tachyzoites in a mouse model of chronic toxoplasmosis. These findings are supported by several previous reports demonstrating that propolis reduced the number of brain cysts in a rat models of chronic toxoplasmosis [135,136]. Furthermore, other authors [104] demonstrated the ability of propolis to improve histological structure and suppress *T. gondii* tachyzoite multiplication. Variations in parasite load reductions could be attributed to differences in animal species, treatment protocols, drug dosages, and the geographical origins of the propolis samples [133,137,138].

Generally, the antiparasitic effects of propolis operate via two mechanisms: directly on the pathogen and indirectly on the host. In the pathogens, propolis can create a physical barrier preventing infiltration into the host cells by blocking essential enzymes and proteins required for invasion [26]. Propolis also disrupts the pathogen’s metabolic processes and reproduction by affecting vital cellular organelles (Figure 6). As an immunomodulator, propolis enhances innate immunity, alters inflammatory signaling pathways, and helps maintain antioxidant status in the host [26,133].

Chagas disease, caused by *T. cruzi*, is a major public health concern worldwide [139,140]. Sousa et al. [141], Reguera-Neto et al. [112], and Salomão et al. [142] reported the trypanocidal potential of green and red propolis ethanolic extracts using the resazurin reduction assay. These extracts inhibited parasitic proliferation with IC_50_ values between 4.3 and 45 μg/mL without damaging host cells. In vivo studies demonstrated that treating infected mice with propolis extracts (25–300 mg/kg body weight/day) for 10 days reduced parasitemia and improved survival without causing liver, muscle, or renal toxicity [142]. Similarly, Dantas et al. [143] showed that treatment with Bulgarian propolis extract decreased intracellular amastigote proliferation in *T. cruzi*-infected skeletal muscle cells.

The antiparasitic activity of a Nigerian propolis extract was evaluated in male albino rats intraperitoneally infected with *T. brucei*. Treatment with 400 and 600 mg/kg doses for five consecutive days significantly reduced parasitemia and increased hematocrit (Hbc) levels and body weight gain [144].

Silva et al. [145] evaluated the effects of Brazilian red propolis against *S. mansoni* in mice infected with immature or adult worms. The propolis extract decreased worm motility and achieved 100% mortality of adult parasites at 25 μg/mL. It also significantly reduced worm burden and egg production during both early and chronic infections.

Sena-Lopes et al. [146] demonstrated in vitro that Brazilian red propolis exhibited effective activity against *T. vaginalis*, inhibiting its growth with an IC_50_ value of 100 μg/mL.

Freitas et al. [147] evaluated the antiparasitic activity of Brazilian propolis extract against *G. duodenalis* trophozoites. Propolis inhibited trophozoite growth with an IC_50_ value of 125 mg/mL and suppressed parasite adherence.

Alday Provencio et al. [148] tested the antiparasitic potential of Mexican propolis extracts against *G. lamblia*, demonstrating a significant inhibition of trophozoite proliferation and viability.

*V. destructor* is an ectoparasite that feeds on the fat body and possibly the hemolymph of honeybees [149], and is considered a major factor in honeybee population decline. [150,151]. Garedew et al. [152] demonstrated the varroacidal activity of German propolis extracts using calorimetric and Petri dish assays. Similar results were reported for Algerian and Argentinean propolis samples at 10% concentration, killing 100% of *Varroa* mites [153,154]. Variability in varroacidal effects is influenced by propolis concentration, contact time, extraction solvent [152], geographical origin, and honeybee species [41,82,155]. Although the exact mechanism remains unclear, it is proposed that propolis weakens the mite’s cuticle, allowing bioactive compounds (phenols and flavonoids) to penetrate [30,156,157] (Figure 6). Additionally, propolis may strengthen honeybees immunity [158]. In mini-hive experiments, spraying with 10% ethanolic propolis extract (40–55% ethanol) eliminated mites without harmless bees [153,154].

#### 4.2.3. Antifungal Activity

Fungal infections are commonly caused by microscopic fungi spread through air, soil, water, and plants, often leading to major losses in food production and agriculture [30]. With the increasing resistance of fungi to chemical fungicides [159], researchers are seeking alternative treatments. Many studies have reported the antifungal activity of propolis against different pathogenic fungi.

Al Ani et al. [101], using a microdilution broth assay, showed that propolis samples from Ireland and the Czech Republic exhibited strong antifungal activity against clinical strains of *Candida glabrata*, *C. parapsilosis*, and *C. tropicalis*, with minimum fungicidal concentrations (MFC) ranging between 0.1 and 5.0 mg/mL. Ota et al. [160] tested 80 *Candida* strains (*C. albicans*, *C. guilliermondii*, *C. krusei*, and *C. tropicalis*) and observed antifungal efficacy in the following order: *C. albicans* > *C. tropicalis* > *C. krusei* > *C. guilliermondii*. Koç et al. [161] tested propolis against 13 strains of *Trichosporon dermatophytes*, with MFC values ranging between 0.0125 and 0.1 µg/mL.

In vitro assays demonstrated that propolis reduced *C. albicans* infection within human dentinal tubules [162]. In another study, 105 human teeth exposed to *C. albicans* for two days and treated with propolis for five days showed inhibition of 99% of fungal growth, comparable to 2% CHX [163]. Moreover, Turkish propolis exhibited antifungal activity similar to chemical fungicides itraconazole and fluconazole against fungal strains isolated from blood [164].

The fungicidal activity of propolis against various fungal species, including *C. albicans*, *C. pelliculosa*, *C. glabrata*, and *C. famata*, has been attributed to the presence of flavonoids, phenolics, and terpenoids [165,166]. Generally, variations in propolis antifungal activity can be influenced by regional flora, climate, seasonal factors [167], as well as differences in the solvent choice, extraction methods, duration, and concentration of bioactive compounds [18].

#### 4.2.4. Antiviral Activity

Viruses are microscopic infectious agents that can only replicate within living host cells and are responsible for various diseases, including the common cold, influenza, smallpox, chickenpox, measles, chronic hepatitis, rabies, AIDS, and COVID-19 [168].

The emergence of these virus infectious diseases has driven researchers to explore natural products as promising sources for new antiviral treatments. Among these, propolis is recognized as a rich reservoir of bioactive compounds with potential therapeutic applications. Numerous studies have described the antiviral properties of propolis using both in vitro and in vivo models against several virus families [21,169]. Gonzalez-Burquez et al. [170] reported that propolis inhibited HIV expression in CD4+ lymphocytes and microglial cell cultures in a concentration-dependent manner. Additionally, Mexican propolis, along with its three purified flavonoids (pinocembrin, naringenin, and quercetin), was evaluated for antiviral activity against canine distemper virus (CDV) in CCL-81 cells. Moreover, coumaric acid and kaempferol, active compounds from Brazilian propolis, demonstrated effectiveness in treating HRV-2, HRV-3, and HRV-4 infections in a HeLa cell culture model [169].

Schnitzler et al. [171] investigated the effects of aqueous and ethanolic propolis extracts on herpes simplex virus type 1 (HSV-1) in cell cultures. Their findings revealed that both extracts significantly inhibited HSV-1 plaque formation by more than 98%. In another study, the antiviral activity of ethanolic propolis extract was assessed against *Varicella zoster* virus (VZV) using a human embryonic lung fibroblast cell line [97]. The results demonstrated significant antiviral efficacy against this virus in viral suspension, with an IC_50_ value of 64 μg/mL, a 94% decrease in viral infectivity. Using the same virus, Labska et al. [172] reported that the antiviral activity of propolis was comparable to that of acyclovir.

An in vivo study involving 90 men and women with recurrent chronic genital herpes simplex virus type 2 (HSV-2) showed that Canadian propolis was more protective than acyclovir and placebo ointments [173]. The replication of HSV-1 and HSV-2 was significantly suppressed in the presence of 25, 50, and 100 μg/mL of Hatay propolis. Moreover, a synergistic effect between Hatay propolis and acyclovir was observed against both HSV-1 and HSV-2 viruses [174].

Recently, during the global COVID-19 pandemic, several studies evaluated the antiviral efficacy of propolis against coronaviruses. Notably, ethanolic extracts of propolis and propolis liposomes were shown to inhibit structural proteins of SARS-CoV-2 in vitro and reduce SARS-CoV-2 infection in Vero E6 cells when used in combination with naringin [175].

To enhance the antiviral potential of propolis, both biotechnological and computational studies have been developed [176]. For instance, liposomal formulations of propolis were tested against SARS-CoV-2 3CL protease and S1 spike protein, revealing that CAPE and rutin exhibited strong binding affinity to both targets. Additionally, the liposomal formulation demonstrated improved antiviral activity compared to standard propolis extract. RT-PCR analysis confirmed that encapsulating propolis extract in liposomes significantly increased its ability to inhibit SARS-CoV-2 replication, achieving effects comparable to those of the antiviral drug remdesivir [177]. Additionally, a study showed that certain active compounds of Algerian propolis exhibited high activity against SARS-CoV-2 main proteases, as determined by molecular docking and ADMET profiling [178]. A preclinical study further revealed that COVID-19 patients treated with green Brazilian propolis experienced faster symptom recovery, while their viral clearance was similar to that of those receiving standard treatment [175]. The authors suggested that several bioactive compounds in propolis contribute to its antiviral activity by disrupting the virion envelope structure or by blocking viral components essential for adsorption and entry into host cells [171]. Furthermore, it was proposed that propolis inhibits virus entry into host cells by blocking key receptors such as ACE2 and TMPRSS2, thus offering protection against viral damage and suppressing replication [169,179]. Additionally, the antiviral potential of propolis has been linked to the modulation of PAK1 signaling pathways and regulation of proinflammatory cytokines, including decreases in TNF-alpha, IL-1 beta, and IL-6 levels [179].

Kwon et al. [169] tested several individual bioactive components of propolis against human rhinoviruses (HRVs) using HeLa cell cultures. Their findings showed that quercetin, kaempferol, luteolin, and chrysin exhibited more pronounced antiviral effects against HRV-2, HRV-3, and HRV-4 compared to the reference drug ribavirin. Additionally, p-coumaric acid and kaempferol demonstrated the ability to inhibit viral infection when administered during the early stages of inoculation, significantly reducing HRV RNA replication in HeLa cell culture. Further, Amoros et al. [180] and Bhargava et al. [30] reported that the synergism between flavonoid compounds may enhance biological activity against various ailments more effectively than individual compounds.

### 4.3. Anti-Inflammatory Activity

Inflammation is a normal process of the innate immune response to environmental changes classified as stress (either exogenous or endogenous), leading to cell or tissue damage and initiated by physical, chemical, and biochemical factors (e.g., parasites, microorganisms, endotoxins) [181]. However, when poorly regulated, inflammation can result in tissue damage and contribute to the development of inflammatory diseases such as asthma, atherosclerosis, cancer, Alzheimer’s disease, and Parkinsonism [181,182,183].

According to the literature, propolis has demonstrated significant anti-inflammatory effects, as evidenced by studies utilizing COX inhibition assays, 5-LOX inhibition assays, and ELISA-based methods. These highlight its potential as a valuable source of anti-inflammatory compounds [184]. However, the cellular and molecular mechanisms by which propolis extracts exhibit an anti-inflammatory activity have not been completely elucidated [100,185].

The main mechanisms underlying the anti-inflammatory effects of propolis involve the inhibition of cyclooxygenase (COX) and lipoxygenase (5-LO) enzymes, which suppress prostaglandin and nitric oxide biosynthesis. Propolis also inhibits the release of arachidonic acid from cell membrane and/or its subsequent metabolism. Furthermore, propolis modulates inflammatory mediators by reducing cytokine levels and exhibiting immunosuppressive activity [186]. Its free radical scavenging activity helps to prevent lipid peroxidation and protects cells from damage induced by free radicals and excessive inflammation [187,188].

These anti-inflammatory properties are largely attributed to the phenolic and flavonoid compounds in propolis, such as CAPE, pinocembrin, chrysin, kaempferol, ferulic acid, caffeic acid, and artepillin-C. These components exhibit strong antioxidant activity and modulate specific inflammation-related signaling pathways [189], including the downregulation of adiponectin mediated by TNF-α and the c-Jun N-terminal kinase (JNK) pathways [186]. They also reduce inflammatory cell infiltration by inhibiting early and late events of T-cell activation and the subsequent release of cytokines [30,190].

Studies by du Toit et al. [191], Jung et al. [192], Cardenas et al. [193], Zhang et al. [194], and Santos et al. [195] reported that flavonoids such as apigenin, luteolin, quercetin, and galangin downregulated inflammatory cytokines, including IL-1β, IL-6, and TNF-α, as well as inflammatory genes such as iNOS, extracellular signal-regulated kinase 1/2 (ERK1/2), and NF-kB. These compounds also inhibit neutrophil migration. Formononetin has been shown to suppress IL-1β activation and cytokine-induced apoptotic signaling by modulating the Bax/Bcl-2 ratio, inhibiting caspase-3 activity, and reducing NO release [196,197]. Szliszka et al. [198] demonstrated that artepillin-C can downregulate inflammatory chemokines such as TNF α, IL-1β, IL-3, IL-4, IL-5, IL-9, IL-12 p40, IL-13, IL-17, G-CSF, GMCSF, MCP-1, MIP-1α, and MIP-1β by modulating the NF-κB pathway.

An in vivo study showed that administering a 200 mg/kg dose of Brazilian propolis extract to mice for 3 days stimulated the innate immunity. This treatment enhanced the immune response by increasing the expression of toll-like receptors TLR-2 and TLR-4, and promoting the production of proinflammatory cytokines IL-2 and IL-6 by macrophages and spleen cells. These effects facilitated microorganism recognition and lymphocyte activation via antigen-presenting cells [199]. Another study confirmed that topical application of Mexican propolis extract improved wound healing in mouse skin due to its strong anti-inflammatory activity [200].

Moreover, the treatment of chronic inflammation in rat models with propolis extract (50–100 mg/kg/day) significantly reduced the arthritis index, confirming its anti-inflammatory efficacy [201]. In a multicenter, double-blinded, randomized, placebo-controlled, parallel-group clinical trial involving 48 patients with rheumatoid arthritis (RA), administration of 500 mg propolis capsules twice daily for 3 months resulted in reduced inflammatory cytokines and oxidative stress, along with improved clinical outcomes. These findings support the need for further trials to evaluate the efficacy of propolis supplementation in RA patients [202].

### 4.4. Immunomodulatory Activity

Several studies, conducted both in vitro and in vivo by researchers worldwide, have highlighted the immunomodulatory effects of propolis [45,203,204]. Chinese propolis has been shown to enhance phagocytic activity and stimulate both cellular and humoral immune responses in mice by increasing serum levels of IgG, IL-1β, IL-4, IL-6, and IFN-γ, as well as promoting splenic lymphocyte proliferation [205]. Another study involving white Roman chickens examined the effects of Chinese propolis on cyclophosphamide (CTX)-induced immunosuppression. It demonstrated that propolis could counteract CTX-induced immunosuppression by significantly enhancing immune organ indexes, promoting lymphocyte proliferation, and elevating serum levels of IL-2 and IL-6 [206].

Using BALB/c male mice, Orsi et al. [207] demonstrated that propolis enhanced IL-1β production and increased the expression of TLR-2 and TLR-4 in peritoneal macrophages, while also upregulating IL-1β and IL-6 production in spleen cells.

The immunomodulatory effects of propolis have also been studied in the context of *Paracoccidioides brasiliensis*, a common systemic mycosis [208]. Since macrophages play a crucial role in host defense, their activation by propolis led to enhanced fungicidal activity [208]. Moreover, Rebouças-Silva et al. [209] evaluated the leishmanicidal and immunomodulatory properties of Brazilian green propolis extract and a gel formulation containing propolis, in both in vitro and in vivo models of *Leishmania amazonensis* infection. In vitro analysis showed that the propolis reduced the infection index of infected macrophages and decreased nitric oxide and prostaglandin E2 levels. In vivo studies revealed that a topical gel containing propolis effectively reduced lesion size in the ears of *L. amazonensis*-infected BALB/c mice after three or seven weeks of treatment, confirming the leishmanicidal and immunomodulatory properties of propolis.

Brazilian and Bulgarian propolis extracts stimulated antibody production in rats immunized with bovine serum albumin, with similar magnitudes observed 15 days post-immunization [210,211]. Furthermore, Doi et al. [212] demonstrated in wild-type and RAG 2-deficient C57BL/6 mice that treatment with 500 mg/kg/day of ethanol propolis extract activated NK cell cytotoxicity.

Mojarab et al. [213] explored the adjuvant potential of propolis extracts combined with a multi-epitope recombinant HIV-1 vaccine in BALB/c mice immunized subcutaneously. The results showed enhanced lymphocyte proliferation, elevated IL-4 and IFN-γ levels, and increased antibody responses, predominantly of the IgG1 subtype. Additionally, the immunostimulatory effects of green Brazilian propolis extract were assessed in combination with an inactivated *SuHV-1* vaccine. The results indicated a significant increase in neutralizing antibody titers and a higher percentage of protected animals upon infection challenge, suggesting that additional co-adjuvants were not necessary [214]. In a follow-up study, the same researchers showed that propolis enhanced cellular immunity by upregulating IFN-γ mRNA expression [215].

**Figure 7:**
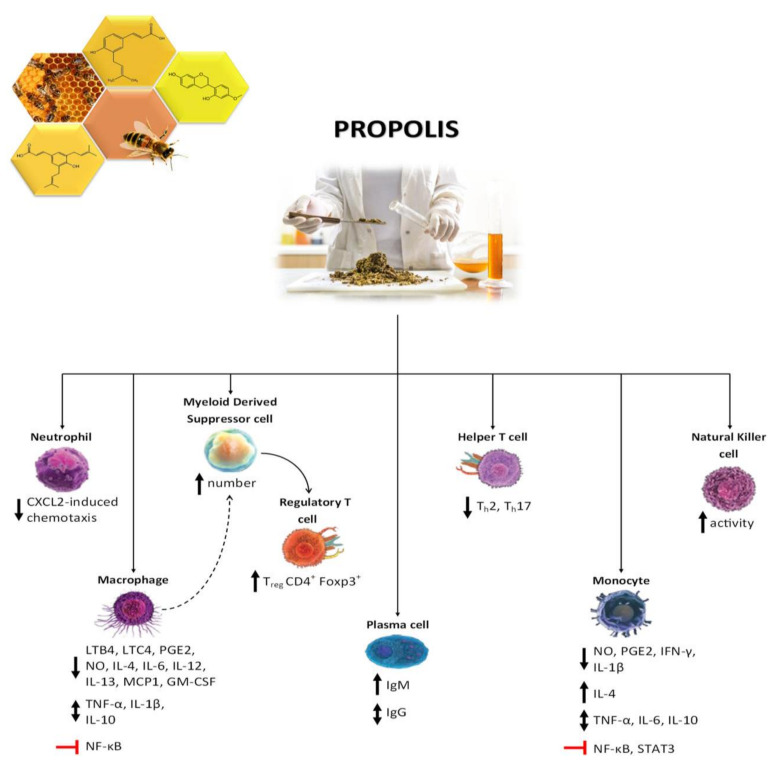
An overview of the immunomodulatory effects by propolis, based on evidence from in vivo and ex vivo experiments. For details, see text. From: Magnavacca et al. [216].

The mechanisms underlying the immunomodulatory effects of propolis have been further elucidated by Magnavacca et al. [216], based on evidence from in vivo and ex vivo experiments (Figure 7).

### 4.5. Antidiabetic Activity

Diabetes is a prevalent chronic endocrine disease characterized by constant hyperglycemia resulting from either insulin deficiency (type 1 DM) or insulin resistance (type 2 DM) [217,218]. In 2021, over 536.6 million individuals worldwide were diagnosed with diabetes. Oxidative stress and excessive production of ROS are major contributors to the development of diabetic complications [219,220], resulting in millions of deaths globally. While various drugs and treatment strategies are used to manage diabetes, many of them cause side-effects—such as stomach pains or diarrhea—which negatively affect patient adherence and therapeutic outcomes [221,222].

The enzymes α-amylase and α-glucosidase play crucial roles in glucose absorption from starch and maltose. Inhibiting α-glucosidase can reduce the hydrolysis of disaccharides, thereby decreasing glucose absorption in the blood [222]. Thus, natural compounds that inhibit these enzymes offer potential as antidiabetic agents.

Several in vitro studies have demonstrated the antidiabetic activity of propolis through α-amylase and α glucosidase inhibition assays [223,224,225]. El Adaouia et al. [223] reported that Turkish ethanolic propolis extract inhibited α-glucosidase and α-amylase with IC_50_ values of 40 and 0.62 μg/mL, respectively. This was supported by Alaribe et al. [226] and El-Guendouz et al. [227], who showed similar effects for Moroccan and Nigerian propolis on and with IC_50_ values ranging between 18 and 55 µg/mL (α-amylase) and 2–7.5 μg/mL (α glucosidase). Chinese propolis also demonstrated strong α-glucosidase inhibition (IC_50_ = 7.2 μg/mL). Vongsak et al. [228] found that Thailand propolis had high inhibitory activity with an IC_50_ of 71 μg/mL, outperforming acarbose (IC_50_ = 156 μg/mL), a standard antidiabetic drug.

In streptozotocin (STZ)-induced diabetic rats, Chinese, Brazilian, and Taiwanese propolis delayed Type-2 diabetes mellitus (T2DM) progression, reduced β-cell failure severity, and prevented hepatorenal damage by reducing lipid peroxidation and enhancing antioxidant enzyme activity [229,230,231].

Propolis from Brazil and Saudi Arabia significantly lowered blood glucose and MDA levels in diabetic rats while increasing renal and serum levels of GSH, SOD, and CAT [229,232]. They also reduced IL-6 levels and reduced immunoglobulin concentrations (IgG, IgA, IgM), improving both biochemical and histological diabetic complications [229,232].

Laaroussi et al. [233] showed that propolis protected against D-glucose-induced T2DM in rats by lowering fasting blood glucose and HbA1c levels, plasma insulin concentrations, and HOMA-IR index, while improving oxidative stress markers in blood, liver, and kidneys. These findings align with previous studies reporting reductions in HbA1c (7.4–8.4%) and total cholesterol levels by 16.6% [234,235,236]. Similarly, Babatunde et al. [237] and Mustafa et al. [238] demonstrated that Chinese and Nigerian propolis improved glycaemia, oxidative stress markers, and protected pancreatic and liver tissues in alloxan-induced diabetic animals.

In a human study, patients with T2DM received either propolis (300 mg), metformin (850 mg), or a placebo twice daily for 12 weeks. Both propolis and metformin significantly reduced FPG, HbA1C, the insulin AUC, and Stumvoll index, while increasing the Matsuda index [239].

### 4.6. Wound Healing Activity

The skin is the largest organ of the human body and serves as first line of defense [240]. When in direct contact with the external environment, it is susceptible to various physical, chemical, and biological stressors, including inflammation and bacterial infections. These disturbances often require prolonged treatment to achieve complete and effective healing [240,241].

Natural wound healing is a complex, multistep process comprising a series of biochemical and cellular events that unfold across four sequential stages: hemostasis (coagulation), inflammation, proliferation of new skin tissue, and remodeling of mature tissue [242,243].

Mast cells play a crucial role in wound healing, being involved in the inflammatory, proliferative, and remodeling phases [204,244,245,246]. During inflammation, mast cells release vasoactive mediators—such as histamine, VEGF, interleukins (IL-6 and IL-8), proteases, TNF-α, and arachidonic acid metabolites [247]—which enhance endothelial permeability, vasodilation, and promote anticoagulant activity. Mast cells accumulate at the wound site, where monocytes and neutrophils release inflammatory mediators, followed by macrophages that facilitate phagocytosis and tissue debridement [241,247,248].

In the proliferative phase, mast cells interact with keratinocytes to promote their migration from the basement membrane into the wound. They also stimulate fibroblasts, which release growth factors essential for tissue regeneration [242,245]. During the remodeling phase, mast cells support angiogenesis, fibroplasia, and re-epithelialization [249]. Propolis has long been used in traditional and complementary medicine to treat wounds and ulcers, employing various mechanisms that offer therapeutic advantages over conventional drugs [250].

Notably, propolis has been shown to act on mast cells, which are critical in all stages of the healing process. Studies have revealed that propolis significantly reduces mast cell numbers during the acute phase of infection—more effectively than dexamethasone [251]. By limiting mast cell proliferation, propolis accelerates healing, reduces inflammation and scaring, and even contributes to keloid formation [252,253]. Its broad-spectrum immunomodulatory [211], antimicrobial [97], antioxidant [254], antiseptic and local anesthetic [243], analgesic and anti-inflammatory [255] properties all contribute to wound healing [256].

Compared to synthetic antimicrobial agents, propolis offers advantages including natural origin, high biocompatibility, and plasticizing properties that improve film flexibility and ease of formulation [25]. Its antimicrobial mechanisms involve disrupting bacterial membrane permeability, inhibiting protein synthesis, and halting cell division [257].

Regarding its anti-inflammatory effects, research shows that propolis enhances innate immunity and regulates inflammatory signaling pathways [23,26]. It inhibits the lipoxygenase pathway in arachidonic acid metabolism [57,258], downregulates iNOS gene expression by targeting its NF-kB promotor site, and directly inhibits iNOS enzymatic activity. Propolis also promotes tumor growth factor β (TGF-β) signaling, reducing the expression of matrix metalloproteinases, proinflammatory cytokines, and eicosanoids, while enhancing Type I collagen deposition [259]. Furthermore, it modulates cellular immune responses by inhibiting macrophage activation and leukocyte recruitment [260,261].

Propolis extract exhibits antioxidant effects by upregulating antioxidant-related genes such as heme oxygenase 1 (HO-1), GCLM, and GCLC in damaged tissue [262]. It also inhibits ROS formation, which limits eicosanoid synthesis, NF-κB activation, inflammatory cytokine expression, and oxidative damage to DNA, RNA, proteins, carbohydrates, and lipids [13]. Additionally, propolis boosts immunity by stimulating interferon production by white blood cells and lymphocytes [22]. These protective effects are attributed to its amino acids, flavonoids, phenolic acids, terpenes, and vitamins [241,250].

Active compounds in propolis—such as chrysin, kaempferol, and CAPE—have been found to reduce mast cell numbers and enhance wound healing during the acute inflammation phase [251]. Those compounds inhibit the release of chemical mediators and inflammatory cytokines, including IL-4, IL-6, and IL-13 [263,264,265,266], and suppress histamine release and the production of other proinflammatory mediators [265,266,267,268].

**Figure 8:**
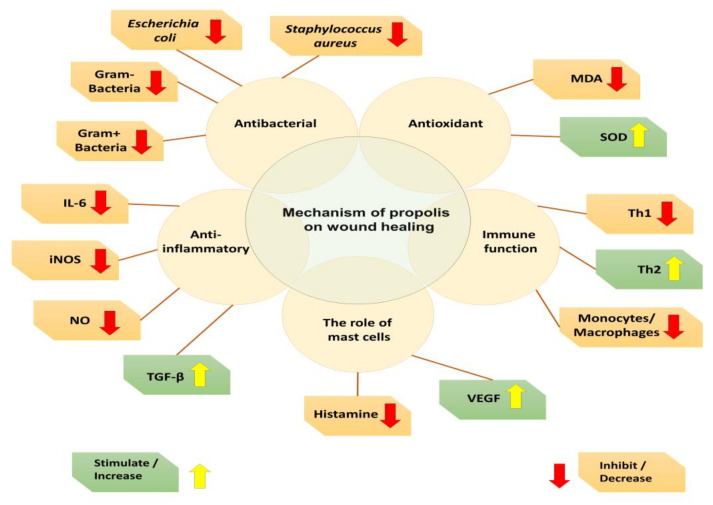
An overview of the wound healing effects of propolis. For detailed mechanisms and supporting evidence, see the main text. Adapted from Yang et al. [241].

Wound healing indicators include collagen deposition, fibrosis, angiogenesis, and infiltration of polymorphonuclear neutrophils. Propolis has been shown to reduce post-healing scarring, enhance wound contraction, shorten healing time [269], and stimulate fibroblast-driven repair by promoting skin cell proliferation, activation, and growth [270]. It also fosters a biochemical environment conducive to re-epithelization [271,272]. Balderas-Cordero et al. [200] reported that propolis can prevent infection, reduce wound contraction time, limit excessive inflammation, and promote the transition from type III to type I collagen—thereby improving tensile strength and accelerating wound closure. Figure 8 summarizes the mechanisms by which propolis facilitates wound healing, as illustrated by Yang et al. [241].

Many authors have demonstrated the high therapeutic efficacy of propolis in various wound types, including burns, diabetes ulcers, oral ulcers, and venous ulcers [273]. In rat model experiments, propolis from different regions of the world was shown to accelerate wound healing across multiple stages of tissue repair, primarily by stimulating keratinocyte proliferation [241,271,274]. This wound healing effect was found to be superior to that of silver sulfadiazine, based on wound healing index scores [275,276].

The effectiveness of propolis in preventing and treating venous ulcers has also been confirmed. In a double blind, randomized clinical trial involving 56 patients with venous leg ulcers, wound healing time was significantly reduced in the propolis-treated group, with complete healing observed by the sixth week [277].

For oral ulcers, the daily administration of 500 mg of propolis over a 6-month period effectively reduced the recurrence of recurrent oral ulcers and improved patients’ quality of life [278].

Regarding diabetic foot ulcers, studies using a type I diabetic mouse model showed that local application of propolis accelerated wound healing and closure. This was achieved through increased production and deposition of type I collagen, reduction in matrix, and decreased inflammation [279].

Clinical trials investigating topical propolis treatment for diabetic foot ulcers revealed that 5% propolis ointment reduced mast cell and neutrophil counts, thereby accelerating tissue regeneration and wound healing [280]. Complete healing of ulcerated areas was observed within 3–7 weeks [281]. Furthermore, Moghtady Khorasgani et al. [276] reported that propolis cream enhanced dermal tissue healing and reduced inflammation more efficiently than silver sulfadiazine.

Another double blind randomized clinical trial evaluated the use of propolis as an adjuvant in diabetic foot ulcers treatment. Results confirmed that propolis promoted wound healing and modulated local inflammation by reducing inflammatory cytokines like TNF-α and increasing anti-inflammatory cytokines such as IL-10 [282,283].

Several studies also demonstrated that propolis accelerates healing of burn wounds by remodeling the extracellular matrix, preventing cell necrosis, and reducing lipid peroxidation [253]. Its antioxidant activity supports its use as a promising agent in burn wound management [284,285,286,287]. In burn wounds, free radical concentrations were lower in propolis-treated sites compared to those treated with silver sulfadiazine [253,279]. Comparative studies by da Rosa et al. [283], Olczyk et al. [288], and Kapare et al. [269] assessed the effectiveness of propolis against silver sulfadiazine and povidone iodine ointment USP (Cipladine^®®^) for burn treatment. The findings confirmed that propolis treatment significantly reduced adhesion protein content, stimulated faster wound healing through collagen deposition, epithelial regeneration, and neovascularization, and decreased inflammation, highlighting its therapeutic potential [289].

An in vivo study in pigs [290], where 72 contact burns were induced, showed that propolis ointment enhanced the rate of collagen extraction more than other preparations. The expression of collagen and its components increased significantly, promoting faster tissue repair [290]. In another in vivo study in rats [291] treated with 5% propolis daily after sustaining second-degree burns on the neck, propolis accelerated tissue repair and reduced local inflammation, demonstrating its successful application in burn healing [291].

### 4.7. Anticancer Activity

Cancer is a major public health concern, with increasing incidence, prevalence, and mortality rates projected for the coming years [292]. It is characterized by uncontrolled cell proliferation and is recognized as a highly heterogeneous and complex disease [30]. Recent studies have highlighted a strong correlation between aging and cancer, as critical tumor suppressor mechanisms regulating the cell cycle are often lost or rendered inactive in cancerous cells [182]. Consequently, aging is considered a pro-tumorigenic state, contributing to cancer progression through physiological changes triggered by stress, DNA damage, and inflammation [29,182]. Current chemotherapeutic and radiotherapeutic treatments are not only highly expensive but associated with significant toxicity and side-effects, including hormonal imbalances, immunosuppression, fatigue, hair loss, and nausea. Moreover, the increasing incidence of drug resistance poses a further challenge to their effectiveness [293,294,295,296]. In light of this, the search for new, naturally derived compounds with anticancer activity and fewer side-effects has intensified in recent years [297,298].

Propolis, historically used for medicinal purposes [167], possesses various biological properties, including antimicrobial, anti-inflammatory, regenerative, immunomodulatory, antioxidant, and antimutagenic effects [7,97,299,300,301]. One of the most extensively studied attributes of propolis is its anticancer effects [302,303,304,305,306]. In this context, propolis and its active compounds have shown promise as adjuncts in cancer treatment and prevention.

Numerous in vitro studies have demonstrated the anticancer activity of propolis from various global regions against multiple cancer cell lines, including human breast cancer (MDA-MB-231, MCF-7, SK-BR-3), bladder cancer (T24), colon cancer (HCT116), embryonic kidney (HEK293), prostate cancer (PC3), ovarian cancer (OVCAR-3), leukemia (K-562), gastric cancer (SGC-7901), lung cancer (A549), liver cancer (HepG2), and pancreatic cancer (PANC1) [307,308,309,310].

The MTT assay, a commonly used method to assess cellular metabolic activity and cytotoxicity, has confirmed the antiproliferative effects of propolis. Results from flow cytometry have shown that propolis induces apoptosis in a dose-dependent manner [311,312,313,314,315,316].

Although the precise mechanisms behind propolis’s anticarcinogenic effects are not fully understood, several studies suggest multiple pathways are involved. These include apoptosis activation [317,318,319], cell cycle regulation [320], immunomodulation [199,321], inhibition of cancer cell migration and invasion [322,323,324], and overcoming drug resistance [325].

Apoptotic effects are mediated via both intrinsic (mitochondrial) and extrinsic (death receptor) pathways, involving increased levels of pro-apoptotic proteins such as p21, Bax, and various phosphorylated forms of p53 (Ser46, Ser15) [29,304]. Intrinsic apoptosis can be triggered by DNA fragmentation, cytokine depletion, or endoplasmic reticulum stress, activating caspases 3 and 7 [326,327]. The extrinsic pathway begins with activation of death receptors on the cell membrane [328]. Additionally, propolis causes cell cycle arrest at various phases (S, G1, G2/M, G0/G1), increases intracellular ROS levels, and disrupts mitochondrial membrane potential [326,329,330,331]. The inflammatory tumor microenvironment plays a key role in cancer progression. Toll-like receptor 4 (TLR4), a critical component of innate immunity, is often dysregulated in cancer, contributing to inflammation, enhanced cell proliferation, and inhibition of apoptosis [332]. Propolis has been shown to stimulate the immune system [333] and inhibit TLR4 signaling, thereby suppressing tumor cell growth [328].

Propolis also exhibits anti-angiogenic effects by interfering with vascular endothelial growth factor (VEGF)-induced vascularization. It inhibits the STAT3 signaling pathway and downregulates MMP-2 and MMP-9, leading to decreased activation of JNK-1 and HIF-1α, thereby reducing angiogenesis and tumor growth [1].

Numerous studies also confirm that propolis inhibits cancer cell migration and invasion in various cancer types, including breast cancer (MCF-7, MDA-MB-231), glioblastoma (U87MG), prostate cancer (DU145, PC3), osteosarcoma (U2OS), fibrosarcoma (HT1080), colorectal cancer (HT-29, LoVo), and lung cancer (A549) [29]. These effects are attributed to modulation of MAPK and PI3K/AKT signaling pathways [334,335,336]. Notably, propolis samples from different global sources have demonstrated anticancer activity with IC_50_ values below 30 μg/mL, a threshold recognized by the U.S. National Cancer Institute as indicating strong cytotoxic potential [337]. Remarkably, propolis has also shown efficacy against cells resistant to standard chemotherapeutic agents [338,339,340].

The antitumor properties of propolis are primarily attributed to its rich composition of flavonoids and phenolic acids. Bioactive compounds such as CAPE, caffeic acid, apigenin, quercetin, genistein, rutin, p-coumaric acid, ferulic acid, kaempferol, naringenin, artepillin C, baccharin, drupanin, cinnamic acid derivatives, prenylated p-coumaric acids, clerodane terpenes, and benzofurans contribute to its anticancer activity [341,342].

Specifically, CAPE inhibits cell proliferation [343] via suppression of p70S6K in the PI3K/AKT pathway [29], induces S-phase arrest and apoptosis [344,345], inhibits migration in MCF-7 and MDA-MB-231 cells, increases LC3-II, and reduces p62 expression to induce autophagy [346]. Chrysin promotes cell cycle arrest and apoptosis via the mitochondrial pathway and enhances ROS production, cytoplasmic Ca^2+^ levels, and lipid peroxidation [347,348,349]. Galangin exerts anticancer effects through multiple signaling pathways and cell cycle arrest [350,351,352,353]. Artepillin C (ARC) shows potent in vitro and in vivo cytotoxicity, inhibiting migration, metastasis, angiogenesis, and matrix metalloproteinase activity [318,354,355]. Apigenin also induces apoptosis and cell cycle arrest [317,356], while cinnamic acid demonstrates antiproliferative activity [308]. In summary, the strong anticancer activity of propolis is believed to result from the synergistic effects and high bioavailability of these phenolic and flavonoid compounds [203,286,297,357,358,359].

Animal studies have confirmed these findings. For example, Benguedouar et al. [351] and Carvalho et al. [360] showed that propolis significantly reduced tumor growth and invasiveness in mice. Inoue et al. [361] found that daily administration of 1400 mg/kg body weight of propolis in mice significantly inhibited tumor growth without toxic effects.

Another in vivo study in rats demonstrated that dietary propolis normalized elevated lactate dehydrogenase (LDH) and transaminase levels induced by MNU, while also boosting antioxidant enzymes such as superoxide dismutase (SOD), catalase (CAT), and glutathione peroxidase (GPx) [362].

In a randomized, double-blind clinical trial involving breast cancer patients undergoing chemotherapy, propolis was shown to exert antioxidant and anti-inflammatory effects [363]. It reduced biomarkers of oxidative stress (carbonyl) and proinflammatory cytokines (TNF-α, IL-2). Propolis also mitigated chemotherapy-induced side effects and enabled higher dosage tolerance [342,364]. Doğan et al. [14] suggested that propolis, in synergy with anticancer agents, enhanced therapeutic efficacy in leukocytes, liver, and kidneys in a dose-dependent manner and reduced the toxicity of radiation and chemotherapeutic treatments.

## 5. Examples of Paramedical Products Based on Propolis

**Figure 9:**
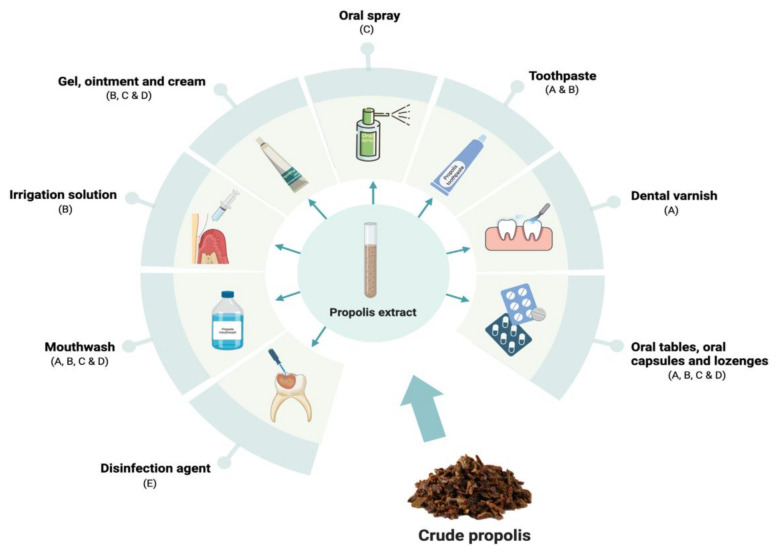
Schematic illustration of different preparations and forms of propolis used in the paramedical field. For details, see text. From: Alghutaimel et al. [368].

Given the well-documented beneficial effects of propolis, new methods of administration are continually being explored and have been the focus of recent drug screening and formulation studies [365,366,367]. In contemporary applications, propolis is incorporated into a variety of products, including throat sprays, skincare formulations, and dietary supplements. Alghutaimel et al. [368] illustrated the diverse preparations and forms of propolis used in paramedical applications (Figure 9).

- Propolis is available in multiple forms, such as tinctures, capsules, sprays, and topical creams. For example, Beelife offers a Green Propolis Extract derived from Brazilian field rosemary, known for its high levels of artepillin-C, a phenolic acid associated with health benefits.

In the medical field, the effectiveness of propolis has been investigated with varying degrees of success for the management of various diseases:

- Beekeeper’s Naturals offers a Propolis Throat Spray aimed at supporting immune health and soothing sore throats. Propolis contains compounds like flavonoids and phenolic acids, which exhibit anti-inflammatory and antimicrobial properties, providing relief from throat irritation and boosting the body’s defense mechanisms against infections [216,369].
- In skincare, propolis is valued for its potential to promote wound healing, reduce acne, and provide antioxidant benefits, making it a popular ingredient in serums and creams. Its antimicrobial and anti-inflammatory properties make it effective for both minor wounds and acne outbreaks. In wound care, propolis accelerates tissue regeneration and reduces infection rates, making it a common component in ointments and creams for cuts, burns, and skin abrasions. Similarly, its antimicrobial effects help prevent the growth of acne-causing bacteria, while its anti-inflammatory properties reduce redness and swelling, promoting faster healing of acne lesions. Propolis extracts have been shown to be particularly effective in treating inflammatory acne and are frequently included in acne treatment serums, gels, and creams [283].
- In dentistry, the antimicrobial effects on oral pathogens, as well as the anti-inflammatory activity of propolis, lead to its use for treating aphthous stomatitis, oral mucositis, acute necrotizing ulcerative gingivitis, pulpitis, gingivitis, and periodontitis. Propolis can be found in toothpaste, mouthwashes, and lozenges, aimed at improving oral hygiene and reducing inflammation and discomfort in the mouth [368].
- In ulcer diseases, propolis may serve as a successful anti-ulcerogenic agent, contributing to the development of novel phytotherapeutic approaches for treating gastric ulcers. Research has indicated that propolis exerts an anti-ulcerogenic effect by reducing gastric acid secretion and enhancing mucosal defense [370,371,372].
- Propolis has been used in veterinary care for treating wounds and infections in animals, as well as enhancing immune responses, although more clinical validation is needed.
- While propolis shows promise in various applications, scientific research on its effectiveness and safety is ongoing [366,373,374]. Individuals with allergies to bee products should exercise caution and consult with a healthcare professional before using propolis-based products.

## 6. Conclusions and Future Perspectives

In conclusion, propolis is a resinous substance produced by honeybees and has garnered significant attention due to its complex composition and diverse biological activities. This review paper presents the hotspots and frontiers of the past 25 years on propolis, delving into its chemical makeup, associated biological functions, current applications, and future prospects.

On the chemical composition, propolis is highly variable, influenced by factors such as geographical location, local flora and bee species, and these can be related to each other. Generally, it comprises resins and vegetable balsams (50%), waxes (30%), essential oils (10%), and pollen (5%). Key constituents include flavonoids, phenolic acids, and their esters, which are primarily responsible for its biological activities. For instance, chrysin, a dihydroxyflavone found in propolis, contributes to its antioxidant properties.

Propolis exhibits a wide range of biological activities, including the following:

- Antimicrobial: Effective against various bacteria, fungi, and viruses, owing to its rich content of flavonoids and phenolics.
- Antioxidant: The presence of polyphenols enables propolis to neutralize free radicals, protecting cells from oxidative stress.
- Anti-inflammatory: Propolis can modulate inflammatory responses, making it beneficial for managing conditions associated with inflammation.
- Immuno-modulatory: Propolis can enhance immune system activity, potentially aiding in the prevention of certain infections.

On current applications, propolis is incorporated into various products across multiple industries:

- Healthcare: Utilized throat sprays, lozenges, and supplements for its antimicrobial and soothing properties. For example, Beekeeper’s Naturals offers a Propolis Throat Spray aimed at supporting immune health.
- Skincare: Valued for its potential to promote wound healing, reduce acne, and provide antioxidant benefits, propolis is a popular ingredient in serums and creams.
- Veterinary medicine: Employed in products designed to enhance animal health due to its antimicrobial and healing properties.

While propolis demonstrates promising therapeutic potential, several considerations are essential for its future use:

- Standardization: The variability in propolis composition necessitates standardized extraction and formulation methods to ensure consistent efficacy and safety.
- Clinical research: Robust clinical trials are required to substantiate the therapeutic claims of propolis and fully understand its mechanisms of action.
- Regulatory frameworks: Establishing clear guidelines will facilitate the safe integration of propolis into mainstream healthcare and consumer products.

In summary, propolis stands out as a multifaceted natural product with a broad spectrum of biological activities. Ongoing research and development are poised to unlock its full potential, paving the way for innovative applications in health and wellness.

## Figures and Tables

**Table 1:** List of keywords and the associated topics included in the literature search, along with the corresponding number of publications retrieved from the PubMed database for papers published between January 2000 and February 2025.

Searched Topics and Keywords	Number of Publications
Propolis	4720
Propolis extraction	2308
Composition of propolis	790
Biological activities of propolis	2307
Application of propolis	665
Formulation of propolis	257

**Table 2:** List of leading institutions (and their countries) involved in propolis research from January 2000 to February 2025, along with their respective key research focus areas.

Name of Institution (Country)	Key Research Focus Areas
University of São Paulo (Brazil)	Brazil is a leading producer of propolis, and the University of São Paulo has been at the forefront of research on its antimicrobial, anti-inflammatory, and antioxidant properties.
Bulgarian Academy of Sciences	The institute has significantly contributed to understanding the chemical profile, diversity and bioactivity of propolis.
University of Zagreb (Croatia)	It is known for studies on the chemical composition and bioactive properties of European propolis.
National Institute of Agricultural Research (INRA) (France)	They have conducted research on the health benefits and applications of propolis in food and medicine.
Chinese Academy of Agricultural Sciences (CAAS) (China)	They have engaged in research on the bioactive components and applications of Chinese propolis.
United States Department of Agriculture (USDA), Arizona, and University of Minnesota (USA)	Researchers at this institution have investigated the microbiota associated with propolis application, enhancing the understanding of its role in honeybee health.

## Data Availability

Not applicable.

## References

[1] Iqbal M., Fan T., Watson D., Alenezi S., Saleh K., Sahlan M. (2019). Preliminary Studies: The Potential Anti-Angiogenic Activities of Two Sulawesi Island (Indonesia) Propolis and Their Chemical Characterization. Heliyon.

[2] Zabaiou N., Fouache A., Trousson A., Buñay-Noboa J., Marceau G., Sapin V., Zellagui A., Baron S., Lahouel M., Lobaccaro J.-M.A. (2019). Ethanolic Extract of Algerian Propolis Decreases Androgen Receptor Transcriptional Activity in Cultured LNCaP Cells. J. Steroid Biochem. Mol. Biol..

[3] Ghisalberti E.L. (1979). Propolis: A Review. Bee World.

[4] Zabaiou N., Fouache A., Trousson A., Baron S., Zellagui A., Lahouel M., Lobaccaro J.-M.A. (2017). Biological Properties of Propolis Extracts: Something New from an Ancient Product. Chem. Phys. Lipids.

[5] Santos L.M., Fonseca M.S., Sokolonski A.R., Deegan K.R., Araújo R.P., Umsza-Guez M.A., Barbosa J.D., Portela R.D., Machado B.A. (2020). Propolis: Types, Composition, Biological Activities, and Veterinary Product Patent Prospecting. J. Sci. Food Agric..

[6] Stojanović S., Najman S.J., Bogdanova-Popov B., Najman S.S. (2020). Propolis: Chemical composition, biological and pharmacological activity-A review. Acta Med. Median..

[7] Khalil M.L. (2006). Biological Activity of Bee Propolis in Health and Disease. Asian Pac. J. Cancer Prev..

[8] Sung S.-H., Choi G.-H., Lee N.-W., Shin B.-C. (2017). External Use of Propolis for Oral, Skin, and Genital Diseases: A Systematic Review and Meta-Analysis. Evid.-Based Complement. Altern. Med..

[9] Havsteen B. (1983). Flavonoids, a Class of Natural Products of High Pharmacological Potency. Biochem. Pharmacol..

[10] Burdock G.A. (1998). Review of the Biological Properties and Toxicity of Bee Propolis (Propolis). Food Chem. Toxicol..

[11] Marcucci M.C. (1995). Propolis: Chemical Composition, Biological Properties and Therapeutic Activity. Apidologie.

[12] Popova M., Giannopoulou E., Skalicka-Woźniak K., Graikou K., Widelski J., Bankova V., Kalofonos H., Sivolapenko G., Gaweł-Bęben K., Antosiewicz B. (2017). Characterization and Biological Evaluation of Propolis from Poland. Molecules.

[13] Martinotti S., Ranzato E. (2015). Propolis: A New Frontier for Wound Healing?. Burn. Trauma.

[14] Doğan H., Silici S., Ozcimen A.A. (2020). Biological Effects of Propolis on Cancer. Turk. J. Agric. Food Sci. Technol..

[15] Huang S., Zhang C.-P., Wang K., Li G., Hu F.-L. (2014). Recent Advances in the Chemical Composition of Propolis. Molecules.

[16] Tani H., Hikami S., Takahashi S., Kimura Y., Matsuura N., Nakamura T., Yamaga M., Koshino H. (2019). Isolation, Identification, and Synthesis of a New Prenylated Cinnamic Acid Derivative from Brazilian Green Propolis and Simultaneous Quantification of Bioactive Components by LC-MS/MS. J. Agric. Food Chem..

[17] Xu X., Yang B., Wang D., Zhu Y., Miao X., Yang W. (2020). The Chemical Composition of Brazilian Green Propolis and Its Protective Effects on Mouse Aortic Endothelial Cells against Inflammatory Injury. Molecules.

[18] Przybyłek I., Karpiński T.M. (2019). Antibacterial Properties of Propolis. Molecules.

[19] Martinello M., Mutinelli F. (2021). Antioxidant Activity in Bee Products: A Review. Antioxidants.

[20] Hotta S., Uchiyama S., Ichihara K. (2020). Brazilian Red Propolis Extract Enhances Expression of Antioxidant Enzyme Genes in Vitro and in Vivo. Biosci. Biotechnol. Biochem..

[21] Nna V.U., Abu Bakar A.B., Md Lazin M.R.M.L., Mohamed M. (2018). Antioxidant, Anti-Inflammatory and Synergistic Anti-Hyperglycemic Effects of Malaysian Propolis and Metformin in Streptozotocin–Induced Diabetic Rats. Food Chem. Toxicol..

[22] Ripari N., Sartori A.A., Da Silva Honorio M., Conte F.L., Tasca K.I., Santiago K.B., Sforcin J.M. (2021). Propolis Antiviral and Immunomodulatory Activity: A Review and Perspectives for COVID-19 Treatment. J. Pharm. Pharmacol..

[23] Franchin M., Freires I.A., Lazarini J.G., Nani B.D., Da Cunha M.G., Colón D.F., De Alencar S.M., Rosalen P.L. (2018). The Use of Brazilian Propolis for Discovery and Development of Novel Anti-Inflammatory Drugs. Eur. J. Med. Chem..

[24] De Mendonça I.C.G., Porto I.C.C.D.M., Do Nascimento T.G., De Souza N.S., Oliveira J.M.D.S., Arruda R.E.D.S., Mousinho K.C., Dos Santos A.F., Basílio-Júnior I.D., Parolia A. (2015). Brazilian Red Propolis: Phytochemical Screening, Antioxidant Activity and Effect against Cancer Cells. BMC Complement. Med. Ther..

[25] Anjum S.I., Ullah A., Khan K.A., Attaullah M., Khan H., Ali H., Bashir M.A., Tahir M., Ansari M.J., Ghramh H.A. (2019). Composition and Functional Properties of Propolis (Bee Glue): A Review. Saudi J. Biol. Sci..

[26] Zulhendri F., Chandrasekaran K., Kowacz M., Ravalia M., Kripal K., Fearnley J., Perera C.O. (2021). Antiviral, Antibacterial, Antifungal, and Antiparasitic Properties of Propolis: A Review. Foods.

[27] Mateo S. (2020). Procédure pour conduire avec succès une revue de littérature selon la méthode PRISMA. Kinésithérapie Rev..

[28] Xiao F., Liu Q., Qin Y., Huang D., Liao Y. (2024). Agricultural Drought Research Knowledge Graph Reasoning by Using VOSviewer. Heliyon.

[29] Forma E., Bryś M. (2021). Anticancer Activity of Propolis and Its Compounds. Nutrients.

[30] Bhargava P., Mahanta D., Kaul A., Ishida Y., Terao K., Wadhwa R., Kaul S.C. (2021). Experimental Evidence for Therapeutic Potentials of Propolis. Nutrients.

[31] Popova M., Bankova V., Butovska D., Petkov V., Nikolova-Damyanova B., Sabatini A.G., Marcazzan G.L., Bogdanov S. (2004). Validated Methods for the Quantification of Biologically Active Constituents of Poplar-type Propolis. Phytochem. Anal..

[32] Righi A.A., Alves T.R., Negri G., Marques L.M., Breyer H., Salatino A. (2011). Brazilian Red Propolis: Unreported Substances, Antioxidant and Antimicrobial Activities. J. Sci. Food Agric..

[33] Piccinelli A.L., Lotti C., Campone L., Cuesta-Rubio O., Campo Fernandez M., Rastrelli L. (2011). Cuban and Brazilian Red Propolis: Botanical Origin and Comparative Analysis by High-Performance Liquid Chromatography–Photodiode Array Detection/Electrospray Ionization Tandem Mass Spectrometry. J. Agric. Food Chem..

[34] Sawaya A.C.H.F., Cunha I.B.S., Marcucci M.C., De Oliveira Rodrigues R.F., Eberlin M.N. (2006). Brazilian Propolis of *Tetragonisca angustula* and *Apis mellifera*. Apidologie.

[35] Alday E., Valencia D., Garibay-Escobar A., Domínguez-Esquivel Z., Piccinelli A.L., Rastrelli L., Monribot-Villanueva J., Guerrero-Analco J.A., Robles-Zepeda R.E., Hernandez J. (2019). Plant Origin Authentication of Sonoran Desert Propolis: An Antiproliferative Propolis from a Semi-Arid Region. Sci. Nat..

[36] Kocot J., Kiełczykowska M., Luchowska-Kocot D., Kurzepa J., Musik I. (2018). Antioxidant Potential of Propolis, Bee Pollen, and Royal Jelly: Possible Medical Application. Oxid. Med. Cell. Longev..

[37] Teixeira É.W., Message D., Negri G., Salatino A., Stringheta P.C. (2010). Seasonal Variation, Chemical Composition and Antioxidant Activity of Brazilian Propolis Samples. Evid.-Based Complement. Altern. Med..

[38] Bankova V.S., De Castro S.L., Marcucci M.C. (2000). Propolis: Recent Advances in Chemistry and Plant Origin. Apidologie.

[39] Bankova V. (2005). Recent Trends and Important Developments in Propolis Research. Evid.-Based Complement. Altern. Med..

[40] Cantarelli M.Á., Camiña J.M., Pettenati E.M., Marchevsky E.J., Pellerano R.G. (2011). Trace Mineral Content of Argentinean Raw Propolis by Neutron Activation Analysis (NAA): Assessment of Geographical Provenance by Chemometrics. LWT Food Sci. Technol..

[41] Silici S., Koç N.A., Ayangil D., Cankaya S. (2005). Antifungal Activities of Propolis Collected by Different Races of Honeybees against Yeasts Isolated from Patients with Superficial Mycoses. J. Pharmacol. Sci..

[42] Marcucci M.C., Ferreres F., Garcίa-Viguera C., Bankova V.S., De Castro S.L., Dantas A.P., Valente P.H.M., Paulino N. (2001). Phenolic Compounds from Brazilian Propolis with Pharmacological Activities. J. Ethnopharmacol..

[43] Guzelmeric E., Ristivojević P., Trifković J., Dastan T., Yilmaz O., Cengiz O., Yesilada E. (2018). Authentication of Turkish Propolis through HPTLC Fingerprints Combined with Multivariate Analysis and Palynological Data and Their Comparative Antioxidant Activity. LWT.

[44] Bozkuş T.N., Değer O., Yaşar A. (2021). Chemical Characterization of Water and Ethanolic Extracts of Turkish Propolis by HPLC-DAD and GC-MS. J. Liq. Chromatogr. Relat. Technol..

[45] Belmehdi O., El Menyiy N., Bouyahya A., El Baaboua A., El Omari N., Gallo M., Montesano D., Naviglio D., Zengin G., Skali Senhaji N. (2023). Recent Advances in the Chemical Composition and Biological Activities of Propolis. Food Rev. Int..

[46] Galeotti F., Maccari F., Fachini A., Volpi N. (2018). Chemical Composition and Antioxidant Activity of Propolis Prepared in Different Forms and in Different Solvents Useful for Finished Products. Foods.

[47] Cottica S.M., Sabik H., Antoine C., Fortin J., Graveline N., Visentainer J.V., Britten M. (2015). Characterization of Canadian Propolis Fractions Obtained from Two-Step Sequential Extraction. LWT Food Sci. Technol..

[48] Jug M., Končić M.Z., Kosalec I. (2014). Modulation of Antioxidant, Chelating and Antimicrobial Activity of Poplar Chemo-Type Propolis by Extraction Procures. LWT Food Sci. Technol..

[49] Taddeo V.A., Epifano F., Fiorito S., Genovese S. (2016). Comparison of Different Extraction Methods and HPLC Quantification of Prenylated and Unprenylated Phenylpropanoids in Raw Italian Propolis. J. Pharm. Biomed. Anal..

[50] Yen C.-H., Chiu H.-F., Wu C.-H., Lu Y.-Y., Han Y.-C., Shen Y.-C., Venkatakrishnan K., Wang C.-K. (2017). Beneficial Efficacy of Various Propolis Extracts and Their Digestive Products by in Vitro Simulated Gastrointestinal Digestion. LWT.

[51] Šuran J., Cepanec I., Mašek T., Radić B., Radić S., Tlak Gajger I., Vlainić J. (2021). Propolis Extract and Its Bioactive Compounds—From Traditional to Modern Extraction Technologies. Molecules.

[52] Bankova V., Trusheva B., Popova M. (2021). Propolis Extraction Methods: A Review. J. Apic. Res..

[53] Pellati F., Prencipe F.P., Bertelli D., Benvenuti S. (2013). An Efficient Chemical Analysis of Phenolic Acids and Flavonoids in Raw Propolis by Microwave-Assisted Extraction Combined with High-Performance Liquid Chromatography Using the Fused-Core Technology. J. Pharm. Biomed. Anal..

[54] Briones-Labarca V., Plaza-Morales M., Giovagnoli-Vicuña C., Jamett F. (2015). High Hydrostatic Pressure and Ultrasound Extractions of Antioxidant Compounds, Sulforaphane and Fatty Acids from Chilean Papaya (*Vasconcellea pubescens*) Seeds: Effects of Extraction Conditions and Methods. LWT Food Sci. Technol..

[55] Chemat F., Rombaut N., Sicaire A.-G., Meullemiestre A., Fabiano-Tixier A.-S., Abert-Vian M. (2017). Ultrasound Assisted Extraction of Food and Natural Products. Mechanisms, Techniques, Combinations, Protocols and Applications. A Review. Ultrason. Sonochem..

[56] Cavalaro R.I., Cruz R.G.D., Dupont S., De Moura Bell J.M.L.N., Vieira T.M.F.D.S. (2019). In Vitro and in Vivo Antioxidant Properties of Bioactive Compounds from Green Propolis Obtained by Ultrasound-Assisted Extraction. Food Chem. X.

[57] Sambou M., Jean-François J., Ndongou Moutombi F.J., Doiron J.A., Hébert M.P.A., Joy A.P., Mai-Thi N.-N., Barnett D.A., Surette M.E., Boudreau L.H. (2020). Extraction, Antioxidant Capacity, 5-Lipoxygenase Inhibition, and Phytochemical Composition of Propolis from Eastern Canada. Molecules.

[58] Reis J.H.D.O., Barreto G.D.A., Cerqueira J.C., Anjos J.P.D., Andrade L.N., Padilha F.F., Druzian J.I., Machado B.A.S. (2019). Evaluation of the Antioxidant Profile and Cytotoxic Activity of Red Propolis Extracts from Different Regions of Northeastern Brazil Obtained by Conventional and Ultrasound-Assisted Extraction. PLoS ONE.

[59] Khacha-ananda S., Tragoolpua K., Chantawannakul P., Tragoolpua Y. (2013). Antioxidant and Anti-Cancer Cell Proliferation Activity of Propolis Extracts from Two Extraction Methods. Asian Pac. J. Cancer Prev..

[60] Ding Q., Sheikh A.R., Gu X., Li J., Xia K., Sun N., Wu R.A., Luo L., Zhang Y., Ma H. (2021). Chinese Propolis: Ultrasound-assisted Enhanced Ethanolic Extraction, Volatile Components Analysis, Antioxidant and Antibacterial Activity Comparison. Food Sci. Nutr..

[61] Corrales M., Toepfl S., Butz P., Knorr D., Tauscher B. (2008). Extraction of Anthocyanins from Grape By-Products Assisted by Ultrasonics, High Hydrostatic Pressure or Pulsed Electric Fields: A Comparison. Innov. Food Sci. Emerg. Technol..

[62] Morelli L.L.L., Prado M.A. (2012). Extraction Optimization for Antioxidant Phenolic Compounds in Red Grape Jam Using Ultrasound with a Response Surface Methodology. Ultrason. Sonochem..

[63] Oliver C.M., Mawson R., Melton L.D., Dumsday G., Welch J., Sanguansri P., Singh T.K., Augustin M.A. (2014). Sequential Low and Medium Frequency Ultrasound Assists Biodegradation of Wheat Chaff by White Rot Fungal Enzymes. Carbohydr. Polym..

[64] Halliwell B., Gutteridge J.M.C. (1984). Oxygen Toxicity, Oxygen Radicals, Transition Metals and Disease. Biochem. J..

[65] Ferreira-Santos P., Genisheva Z., Botelho C., Rocha C., António Teixeira J., Waisundara V. (2021). Valorization of Natural Antioxidants for Nutritional and Health Applications. Antioxidants-Benefits, Sources, Mechanisms of Action.

[66] Xiao F., Xu T., Lu B., Liu R. (2020). Guidelines for Antioxidant Assays for Food Components. Food Front..

[67] Alam M.N., Bristi N.J., Rafiquzzaman M. (2013). Review on in Vivo and in Vitro Methods Evaluation of Antioxidant Activity. Saudi Pharm. J..

[68] Singh S., Singh R.P. (2008). In Vitro Methods of Assay of Antioxidants: An Overview. Food Rev. Int..

[69] Sun C., Wu Z., Wang Z., Zhang H. (2015). Effect of Ethanol/Water Solvents on Phenolic Profiles and Antioxidant Properties of Beijing Propolis Extracts. Evid.-Based Complement. Altern. Med..

[70] Bittencourt M.L.F., Ribeiro P.R., Franco R.L.P., Hilhorst H.W.M., De Castro R.D., Fernandez L.G. (2015). Metabolite Profiling, Antioxidant and Antibacterial Activities of Brazilian Propolis: Use of Correlation and Multivariate Analyses to Identify Potential Bioactive Compounds. Food Res. Int..

[71] Narimane S., Demircan E., Salah A., Ozcelik B.Ö., Salah R. (2017). Correlation between Antioxidant Activity and Phenolic Acids Profile and Content of Algerian Propolis: Influence of Solvent. Pak. J. Pharm. Sci..

[72] Andrade J.K.S., Denadai M., De Oliveira C.S., Nunes M.L., Narain N. (2017). Evaluation of Bioactive Compounds Potential and Antioxidant Activity of Brown, Green and Red Propolis from Brazilian Northeast Region. Food Res. Int..

[73] Zhang C., Shen X., Chen J., Jiang X., Hu F. (2017). Identification of Free Radical Scavengers from Brazilian Green Propolis Using Off-Line HPLC-DPPH Assay and LC-MS. J. Food Sci..

[74] Hegazi A.G., Abd El Hady F.K., Abd Allah F.A.M. (2000). Chemical Composition and Antimicrobial Activity of European Propolis. Z. Naturforsch. C.

[75] Naik R.R., Shakya A.K., Oriquat G.A., Katekhaye S., Paradkar A., Fearnley H., Fearnley J. (2021). Fatty Acid Analysis, Chemical Constituents, Biological Activity and Pesticide Residues Screening in Jordanian Propolis. Molecules.

[76] Do Nascimento T.G., Da Silva P.F., Azevedo L.F., Da Rocha L.G., De Moraes Porto I.C.C., Lima E Moura T.F.A., Basílio-Júnior I.D., Grillo L.A.M., Dornelas C.B., Fonseca E.J.D.S. (2016). Polymeric Nanoparticles of Brazilian Red Propolis Extract: Preparation, Characterization, Antioxidant and Leishmanicidal Activity. Nanoscale Res. Lett..

[77] Zhang H., Fu Y., Niu F., Li Z., Ba C., Jin B., Chen G., Li X. (2018). Enhanced Antioxidant Activity and in Vitro Release of Propolis by Acid-Induced Aggregation Using Heat-Denatured Zein and Carboxymethyl Chitosan. Food Hydrocoll..

[78] Benković V., Orsolić N., Knežević A.H., Ramić S., Ðikić D., Bašić I., Kopjar N. (2008). Evaluation of the Radioprotective Effects of Propolis and Flavonoids in Gamma-Irradiated Mice: The Alkaline Comet Assay Study. Biol. Pharm. Bull..

[79] Abdullah N.A., Zullkiflee N., Zaini S.N.Z., Taha H., Hashim F., Usman A. (2020). Phytochemicals, Mineral Contents, Antioxidants, and Antimicrobial Activities of Propolis Produced by Brunei Stingless Bees *Geniotrigona thoracica*, *Heterotrigona itama*, and *Tetrigona binghami*. Saudi J. Biol. Sci..

[80] Daleprane J.B., Abdalla D.S. (2013). Emerging Roles of Propolis: Antioxidant, Cardioprotective, and Antiangiogenic Actions. Evid.-Based Complement. Altern. Med..

[81] El-ghazaly M.A., Abd El-Naby D.H., Khayyal M.T. (2011). The Influence of Irradiation on the Potential Chondroprotective Effect of Aqueous Extract of Propolis in Rats. Int. J. Radiat. Biol..

[82] Laaroussi H., Ferreira-Santos P., Genisheva Z., Bakour M., Ousaaid D., Teixeira J.A., Lyoussi B. (2021). Unraveling the Chemical Composition, Antioxidant, α-Amylase and α-Glucosidase Inhibition of Moroccan Propolis. Food Biosci..

[83] Barrientos L., Herrera C.L., Montenegro G., Ortega X., Veloz J., Alvear M., Cuevas A., Saavedra N., Salazar L.A. (2013). Chemical and Botanical Characterization of Chilean Propolis and Biological Activity on Cariogenic Bacteria *Streptococcus Mutans* and *Streptococcus Sobrinus*. Braz. J. Microbiol..

[84] Devequi-Nunes D., Machado B.A.S., Barreto G.D.A., Rebouças Silva J., Da Silva D.F., Da Rocha J.L.C., Brandão H.N., Borges V.M., Umsza-Guez M.A. (2018). Chemical Characterization and Biological Activity of Six Different Extracts of Propolis through Conventional Methods and Supercritical Extraction. PLoS ONE.

[85] Johnston J.E., Sepe H.A., Miano C.L., Brannan R.G., Alderton A.L. (2005). Honey Inhibits Lipid Oxidation in Ready-to-Eat Ground Beef Patties. Meat Sci..

[86] Küçük M., Kolaylı S., Karaoğlu Ş., Ulusoy E., Baltacı C., Candan F. (2007). Biological Activities and Chemical Composition of Three Honeys of Different Types from Anatolia. Food Chem..

[87] Bankova V., Bertelli D., Borba R., Conti B.J., Da Silva Cunha I.B., Danert C., Eberlin M.N., I Falcão S., Isla M.I., Moreno M.I.N. (2019). Standard Methods for *Apis Mellifera* Propolis Research. J. Apic. Res..

[88] Bonamigo T., Campos J.F., Oliveira A.S., Torquato H.F.V., Balestieri J.B.P., Cardoso C.A.L., Paredes-Gamero E.J., De Picoli Souza K., Dos Santos E.L. (2017). Antioxidant and Cytotoxic Activity of Propolis of *Plebeia droryana* and *Apis mellifera* (Hymenoptera, Apidae) from the Brazilian Cerrado Biome. PLoS ONE.

[89] Calegari M.A., Prasniewski A., Silva C.D., Sado R.Y., Maia F.M.C., Tonial L.M.S., Oldoni T.L.C. (2017). Propolis from Southwest of Parana Produced by Selected Bees: Influence of Seasonality and Food Supplementation on Antioxidant Activity and Phenolic Profile. An. Acad. Bras. Ciências.

[90] De Lima G.G., De Souza R.O., Bozzi A.D., Poplawska M.A., Devine D.M., Nugent M.J.D. (2016). Extraction Method Plays Critical Role in Antibacterial Activity of Propolis-Loaded Hydrogels. J. Pharm. Sci..

[91] De Zordi N., Cortesi A., Kikic I., Moneghini M., Solinas D., Innocenti G., Portolan A., Baratto G., Dall’Acqua S. (2014). The Supercritical Carbon Dioxide Extraction of Polyphenols from Propolis: A Central Composite Design Approach. J. Supercrit. Fluids.

[92] Lopes A.A., Ferreira T.S., Nesi R.T., Lanzetti M., Pires K.M.P., Silva A.M., Borges R.M., Silva A.J.R., Valença S.S., Porto L.C. (2013). Antioxidant Action of Propolis on Mouse Lungs Exposed to Short-Term Cigarette Smoke. Bioorganic Med. Chem..

[93] Da Silveira C.C.S.D.M., Fernandes L.M.P., Silva M.L., Luz D.A., Gomes A.R.Q., Monteiro M.C., Machado C.S., Torres Y.R., Lira T.O.D., Ferreira A.G. (2016). Neurobehavioral and Antioxidant Effects of Ethanolic Extract of Yellow Propolis. Oxid. Med. Cell. Longev..

[94] Sun F., Hayami S., Haruna S., Ogiri Y., Tanaka K., Yamada Y., Ikeda K., Yamada H., Sugimoto H., Kawai N. (2000). In Vivo Antioxidative Activity of Propolis Evaluated by the Interaction with Vitamins C and E and the Level of Lipid Hydroperoxides in Rats. J. Agric. Food Chem..

[95] Remirez D., González R., Rodriguez S., Ancheta O., Bracho J.C., Rosado A., Rojas E., Ramos M.E. (1997). Protective Effects of Propolis Extract on Allyl Alcohol-Induced Liver Injury in Mice. Phytomedicine.

[96] De Lima R.O.A., Bazo A.P., Said R.A., Sforcin J.M., Bankova V., Darros B.R., Salvadori D.M.F. (2005). Modifying Effect of Propolis on Dimethylhydrazine-Induced DNA Damage but Not Colonic Aberrant Crypt Foci in Rats. Environ. Mol. Mutagen..

[97] Nedji N., Loucif-Ayad W. (2014). Antimicrobial Activity of Algerian Propolis in Foodborne Pathogens and Its Quantitative Chemical Composition. Asian Pac. J. Trop. Dis..

[98] Vică M.L., Glevitzky M., Tit D.M., Behl T., Heghedűş-Mîndru R.C., Zaha D.C., Ursu F., Popa M., Glevitzky I., Bungău S. (2021). The Antimicrobial Activity of Honey and Propolis Extracts from the Central Region of Romania. Food Biosci..

[99] Nandre V.S., Bagade A.V., Kasote D.M., Lee J.H.J., Kodam K.M., Kulkarni M.V., Ahmad A. (2021). Antibacterial Activity of Indian Propolis and Its Lead Compounds against Multi-Drug Resistant Clinical Isolates. J. Herb. Med..

[100] Belmehdi O., Bouyahya A., Jekő J., Cziáky Z., Zengin G., Sotkó G., El Baaboua A., Skali Senhaji N., Abrini J. (2021). Chemical Analysis, Antibacterial, and Antioxidant Activities of Flavonoid-rich Extracts from Four Moroccan Propolis. J. Food Process. Preserv..

[101] AL-Ani I., Zimmermann S., Reichling J., Wink M. (2018). Antimicrobial Activities of European Propolis Collected from Various Geographic Origins Alone and in Combination with Antibiotics. Medicines.

[102] Kahraman-Ilıkkan Ö. (2023). Bacterial Profile and Fatty Acid Composition of Anatolian Bee Bread Samples by Metataxonomic and Metabolomic Approach. Curr. Microbiol..

[103] Nazeri R., Ghaiour M., Abbasi S. (2019). Evaluation of Antibacterial Effect of Propolis and Its Application in Mouthwash Production. Front. Dent..

[104] Hegazi A.G., El-Fadaly H.A., Barakat A.M., Abou-El-Doubal S.K.A. (2015). In Vitro Effects of Some Bee Products on *T. gondii* Tachyzoites. Glob. Vet..

[105] Weinstein M.P. (2020). Performance Standards for Antimicrobial Susceptibility Testing.

[106] Boufadi Y.M., Soubhye J., Riazi A., Rousseau A., Vanhaeverbeek M., Nève J., Boudjeltia K.Z., Van Antwerpen P. (2014). Characterization and Antioxidant Properties of Six Algerian Propolis Extracts: Ethyl Acetate Extracts Inhibit Myeloperoxidase Activity. Int. J. Mol. Sci..

[107] Seidel V., Peyfoon E., Watson D.G., Fearnley J. (2008). Comparative Study of the Antibacterial Activity of Propolis from Different Geographical and Climatic Zones. Phytother. Res..

[108] Muli E.M., Maingi J.M. (2007). Antibacterial Activity of *Apis Mellifera L.* Propolis Collected in Three Regions of Kenya. J. Venom. Anim. Toxins Incl. Trop. Dis..

[109] Mohan P.V.M.U., Uloopi K., Vinay C., Rao R. (2016). In Vivo Comparison of Cavity Disinfection Efficacy with APF Gel, Propolis, Diode Laser, and 2% Chlorhexidine in Primary Teeth. Contemp. Clin. Dent..

[110] Falcão S.I., Vale N., Cos P., Gomes P., Freire C., Maes L., Vilas-Boas M. (2014). In Vitro Evaluation of Portuguese Propolis and Floral Sources for Antiprotozoal, Antibacterial and Antifungal Activity. Phytother. Res..

[111] Xie X.-L., Gi M., Fujioka M., Doi K., Yamano S., Tachibana H., Fang H., Kakehashi A., Wanibuchi H. (2015). Ethanol-Extracted Propolis Enhances BBN-Initiated Urinary Bladder Carcinogenesis via Non-Mutagenic Mechanisms in Rats. Food Chem. Toxicol..

[112] Regueira-Neto M.D.S., Tintino S.R., Rolón M., Coronal C., Vega M.C., De Queiroz Balbino V., De Melo Coutinho H.D. (2018). Antitrypanosomal, Antileishmanial and Cytotoxic Activities of Brazilian Red Propolis and Plant Resin of *Dalbergia ecastaphyllum (L)* Taub. Food Chem. Toxicol..

[113] Velikova M., Bankova V., Sorkun K., Houcine S., Tsvetkova I., Kujumgiev A. (2000). Propolis from the Mediterranean Region: Chemical Composition and Antimicrobial Activity. Z. Naturforsch. C.

[114] Popova M., Silici S., Kaftanoglu O., Bankova V. (2005). Antibacterial Activity of Turkish Propolis and Its Qualitative and Quantitative Chemical Composition. Phytomedicine.

[115] Afrouzan H., Zakeri S., Abouie Mehrizi A., Molasalehi S., Tahghighi A., Shokrgozar M.A., Es-Haghi A., Dinparast Djadid N. (2017). Anti-Plasmodial Assessment of Four Different Iranian Propolis Extracts. Arch. Iran. Med..

[116] Boufadi Y.M., Soubhye J., Nève J., Van Antwerpen P., Riazi A. (2016). Antimicrobial Effects of Six Algerian Propolis Extracts. Int. J. Food Sci. Technol..

[117] Kartal M., Yildiz S., Kaya S., Kurucu S., Topçu G. (2003). Antimicrobial Activity of Propolis Samples from Two Different Regions of Anatolia. J. Ethnopharmacol..

[118] Stepanović S., Antić N., Dakić I., Svabić-Vlahović M. (2003). In Vitro Antimicrobial Activity of Propolis and Synergism between Propolis and Antimicrobial Drugs. Microbiol. Res..

[119] Astani A., Zimmermann S., Hassan E., Reichling J., Sensch K.H., Schnitzler P. (2013). Antimicrobial Activity of Propolis Special Extract GH 2002 against Multidrug-Resistant Clinical Isolates. Pharmazie.

[120] Boyanova L., Kolarov R., Gergova G., Mitov I. (2006). In Vitro Activity of Bulgarian Propolis against 94 Clinical Isolates of Anaerobic Bacteria. Anaerobe.

[121] De Souza Silva T., Silva J.M.B., Braun G.H., Mejia J.A.A., Ccapatinta G.V.C., Santos M.F.C., Tanimoto M.H., Bastos J.K., Parreira R.L.T., Orenha R.P. (2021). Green and Red Brazilian Propolis: Antimicrobial Potential and Anti-Virulence against ATCC and Clinically Isolated Multidrug-Resistant Bacteria. Chem. Biodivers..

[122] Ratajczak M., Kaminska D., Matuszewska E., Hołderna-Kedzia E., Rogacki J., Matysiak J. (2021). Promising Antimicrobial Properties of Bioactive Compounds from Different Honeybee Products. Molecules.

[123] Al-Waili N., Al-Ghamdi A., Ansari M.J., Al-Attal Y., Salom K. (2012). Synergistic Effects of Honey and Propolis toward Drug Multi-Resistant *Staphylococcus aureus*, *Escherichia coli* and *Candida albicans* Isolates in Single and Polymicrobial Cultures. Int. J. Med. Sci..

[124] Wojtyczka R.D., Dziedzic A., Idzik D., Kępa M., Kubina R., Kabała-Dzik A., Smoleń-Dzirba J., Stojko J., Sajewicz M., Wąsik T.J. (2013). Susceptibility of *Staphylococcus aureus* Clinical Isolates to Propolis Extract Alone or in Combination with Antimicrobial Drugs. Molecules.

[125] Orsi R.O., Fernandes A., Bankova V., Sforcin J.M. (2012). The Effects of Brazilian and Bulgarian Propolis in Vitro against *Salmonella* Typhi and Their Synergism with Antibiotics Acting on the Ribosome. Nat. Prod. Res..

[126] Kalia P., Kumar N.R., Harjai K. (2016). Studies on the Therapeutic Effect of Propolis along with Standard Antibacterial Drug in *Salmonella enterica* serovar Typhimurium Infected BALB/c Mice. BMC Complement. Altern. Med..

[127] Santiago K.B., Piana G.M., Conti B.J., Cardoso E.D.O., Murbach Teles Andrade B.F., Zanutto M.R., Mores Rall V.L., Fernandes A., Sforcin J.M. (2018). Microbiological Control and Antibacterial Action of a Propolis-Containing Mouthwash and Control of Dental Plaque in Humans. Nat. Prod. Res..

[128] Bazvand L., Aminozarbian M.G., Farhad A., Noormohammadi H., Hasheminia S.M., Mobasherizadeh S. (2014). Antibacterial Effect of Triantibiotic Mixture, Chlorhexidine Gel, and Two Natural Materials Propolis and Aloe Vera against *Enterococcus faecalis*: An Ex Vivo Study. Dent. Res. J..

[129] Carbajal Mejía J.B. (2014). Antimicrobial Effects of Calcium Hydroxide, Chlorhexidine, and Propolis on *Enterococcus faecalis* and *Candida albicans*. J. Investig. Clin. Dent..

[130] Anauate-Netto C., Anido-Anido A., Lewgoy H.R., Matsumoto R., Alonso R.C.B., Marcucci M.C., Paulino N., Bretz W.A. (2014). Randomized, Double-Masked, Placebo-Controlled Clinical Trial on the Effects of Propolis and Chlorhexidine Mouthrinses on Gingivitis. Braz. Dent. Sci..

[131] Vasconcelos W.A., Braga N.M.A., Chitarra V.R., Santos V.R., Andrade Â.L., Domingues R.Z. (2014). Bioactive Glass-Green and Red Propolis Association: Antimicrobial Activity Against Oral Pathogen Bacteria. Nat. Prod. Chem. Res..

[132] Ramos J.M., Milla A., Rodríguez J.C., Padilla S., Masiá M., Gutiérrez F. (2011). Seroprevalence of *Toxoplasma gondii* Infection among Immigrant and Native Pregnant Women in Eastern Spain. Parasitol. Res..

[133] Dwivedi K., Mandal A.K., Afzal O., Altamimi A.S.A., Sahoo A., Alossaimi M.A., Almalki W.H., Alzahrani A., Barkat M.A., Almeleebia T.M. (2023). Emergence of Nano-Based Formulations for Effective Delivery of Flavonoids against Topical Infectious Disorders. Gels.

[134] Elmahallawy E.K., El Fadaly H.A.M., Soror A.H., Ali F.A.Z., Abd El-Razik K.A., Soliman Y.A., Alkhaldi A.A.M., Albezrah N.K.A., Barakat A.M. (2022). Novel Insights on the Potential Activity of Propolis and Wheat Germ Oil against Chronic Toxoplasmosis in Experimentally Infected Mice. Biomed. Pharmacother..

[135] Hegazi A., Toaleb N., El Fadaly H.A., Abdel-Rahman E.H., Barakat A.M. (2021). In Vivo -Cellular and Humoral Immune Response for Evaluation of Propolis Effect on Chronic Toxoplasmosis in Rats. Adv. Anim. Vet. Sci..

[136] Hagras N.A., Mogahed N.M.F.H., Sheta E., Darwish A.A., El-hawary M.A., Hamed M.T., Elwakil B.H. (2022). The Powerful Synergistic Effect of Spiramycin/Propolis Loaded Chitosan/Alginate Nanoparticles on Acute Murine Toxoplasmosis. PLOS Neglected Trop. Dis..

[137] Al Nasr I., Ahmed F., Pullishery F., El-Ashram S., Ramaiah V.V. (2016). Toxoplasmosis and Anti-Toxoplasma Effects of Medicinal Plant Extracts-A Mini-Review. Asian Pac. J. Trop. Med..

[138] Konstantinovic N., Guegan H., Stäjner T., Belaz S., Robert-Gangneux F. (2019). Treatment of Toxoplasmosis: Current Options and Future Perspectives. Food Waterborne Parasitol..

[139] Sousa L., Azevedo M.L., Rocha D., Andrade Â., Amparo T., Dos Santos O., Seibert J., Pereira L., Vieira P., Carneiro C. (2021). Trypanocidal Activity and Increased Solubility of Benznidazole Incorporated in PEG 4000 and Its Derivatives. J. Braz. Chem. Soc..

[140] Morel C.M. (1999). Chagas Disease, from Discovery to Control-and beyond: History, Myths and Lessons to Take Home. Mem. Inst. Oswaldo Cruz.

[141] Sousa L.R.D., Amparo T.R., de Souza G.H.B., Ferraz A.T., Fonseca K.S., de Azevedo A.S., Nascimento A.M.D., Andrade Â.L., Seibert J.B., Valverde T.M. (2023). Anti-Trypanosoma Cruzi Potential of Vestitol Isolated from Lyophilized Red Propolis. Molecules.

[142] Salomão K., De Souza E.M., Henriques-Pons A., Barbosa H.S., De Castro S.L. (2011). Brazilian Green Propolis: Effects In Vitro and In Vivo on *Trypanosoma cruzi*. Evid.-Based Complement. Altern. Med..

[143] Dantas A.P., Salomão K., Barbosa H.S., De Castro S.L. (2006). The Effect of Bulgarian Propolis against *Trypanosoma Cruzi* and during Its Interaction with Host Cells. Mem. Inst. Oswaldo Cruz.

[144] Nweze N.E., Okoro H.O., Al Robaian M., Omar R.M.K., Tor-Anyiin T.A., Watson D.G., Igoli J.O. (2017). Effects of Nigerian Red Propolis in Rats Infected with *Trypanosoma brucei brucei*. Comp. Clin. Pathol..

[145] Silva M.P., Silva T.M., Mengarda A.C., Salvadori M.C., Teixeira F.S., Alencar S.M., Luz Filho G.C., Bueno-Silva B., De Moraes J. (2021). Brazilian Red Propolis Exhibits Antiparasitic Properties in Vitro and Reduces Worm Burden and Egg Production in an Mouse Model Harboring Either Early or Chronic Schistosoma Mansoni Infection. J. Ethnopharmacol..

[146] Sena-Lopes Â., Bezerra F.S.B., Das Neves R.N., De Pinho R.B., Silva M.T.D.O., Savegnago L., Collares T., Seixas F., Begnini K., Henriques J.A.P. (2018). Chemical Composition, Immunostimulatory, Cytotoxic and Antiparasitic Activities of the Essential Oil from Brazilian Red Propolis. PLoS ONE.

[147] Freitas S.F., Shinohara L., Sforcin J.M., Guimarães S. (2006). In Vitro Effects of Propolis on *Giardia* duodenalis trophozoites. Phytomedicine.

[148] Alday-Provencio S., Diaz G., Rascon L., Quintero J., Alday E., Robles-Zepeda R., Garibay-Escobar A., Astiazaran H., Hernandez J., Velazquez C. (2015). Sonoran Propolis and Some of Its Chemical Constituents Inhibit In Vitro Growth of *Giardia lamblia* trophozoites. Planta Medica.

[149] Ramsey S.D., Ochoa R., Bauchan G., Gulbronson C., Mowery J.D., Cohen A., Lim D., Joklik J., Cicero J.M., Ellis J.D. (2019). *Varroa destructor* Feeds Primarily on HoneyBee Fat Body Tissue and Not Hemolymph. Proc. Natl. Acad. Sci. USA.

[150] Orantes-Bermejo F.J., Pajuelo A.G., Megías M.M., Fernández-Píñar C.T. (2010). Pesticide Residues in Beeswax and Beebread Samples Collected from HoneyBee Colonies (*Apis mellifera* L.) in Spain. Possible Implications for Bee Losses. J. Apic. Res..

[151] Nazzi F., Brown S.P., Annoscia D., Del Piccolo F., Di Prisco G., Varricchio P., Della Vedova G., Cattonaro F., Caprio E., Pennacchio F. (2012). Synergistic Parasite-Pathogen Interactions Mediated by Host Immunity Can Drive the Collapse of Honeybee Colonies. PLoS Pathog..

[152] Garedew A., Lamprecht I., Schmolz E., Schricker B. (2002). The Varroacidal Action of Propolis: A Laboratory Assay. Apidologie.

[153] Damiani N., Fernández N.J., Maldonado L.M., Alvarez A.R., Eguaras M.J., Marcangeli J.A. (2010). Bioactivity of Propolis from Different Geographical Origins on *Varroa destructor* (Acari: Varroidae). Parasitol. Res..

[154] Ayad A.S., Benchaabane S., Daas T., Smagghe G., Loucif-Ayad W. (2024). Assessment of Efficacy of Algerian Propolis against the Parasitic Mite *Varroa destructor* and Safety for HoneyBees by Spray Treatment. Insects.

[155] El Menyiy N., Bakour M., El Ghouizi A., El Guendouz S., Lyoussi B. (2021). Influence of Geographic Origin and Plant Source on Physicochemical Properties, Mineral Content, and Antioxidant and Antibacterial Activities of Moroccan Propolis. Int. J. Food Sci..

[156] Drescher N., Klein A.-M., Neumann P., Yañez O., Leonhardt S. (2017). Inside Honeybee Hives: Impact of Natural Propolis on the Ectoparasitic Mite *Varroa destructor* and Viruses. Insects.

[157] Damiani N., Maggi M.D., Gende L.B., Faverin C., Eguaras M.J., Marcangeli J.A. (2010). Evaluation of the Toxicity of a Propolis Extract on *Varroa destructor* (Acari: Varroidae) and *Apis mellifera* (Hymenoptera: Apidae). J. Apic. Res..

[158] Evans J.D., Aronstein K., Chen Y.P., Hetru C., Imler J.-L., Jiang H., Kanost M., Thompson G.J., Zou Z., Hultmark D. (2006). Immune Pathways and Defence Mechanisms in Honey Bees *Apis mellifera*. Insect Mol. Biol..

[159] Rex J.H., Rinaldi M.G., Pfaller M.A. (1995). Resistance of *Candida* Species to Fluconazole. Antimicrob. Agents Chemother..

[160] Ota C., Unterkircher C., Fantinato V., Shimizu M.T. (2001). Antifungal Activity of Propolis on Different Species of *Candida*. Mycoses.

[161] Koç A.N., Silici S., Kasap F., Hörmet-Öz H.T., Mavus-Buldu H., Ercal B.D. (2011). Antifungal Activity of the Honeybee Products Against *Candida* spp. and *Trichosporon* spp. J. Med. Food.

[162] Chua E.G., Parolia A., Ahlawat P., Pau A., Amalraj F.D. (2014). Antifungal Effectiveness of Various Intracanal Medicaments against *Candida albicans*: An Ex-Vivo Study. BMC Oral Health.

[163] Joy Sinha D., Garg P., Verma A., Malik V., Maccune E.R., Vasudeva A. (2015). Dentinal Tubule Disinfection with Propolis & Two Extracts of Azadirachta Indica Against *Candida albicans* Biofilm Formed on Tooth Substrate. Open Dent. J..

[164] Mutlu Sariguzel F., Berk E., Koc A.N., Sav H., Demir G. (2016). Antifungal Activity of Propolis Against Yeasts Isolated from Blood Culture: In Vitro Evaluation. Clin. Lab. Anal..

[165] Correa L., de Carvalho Meirelles G., Balestrin L., de Souza P.O., Moreira J.C.F., Schuh R.S., Bidone J., von Poser G.L., Teixeira H.F. (2020). In Vitro Protective Effect of Topical Nanoemulgels Containing Brazilian Red Propolis Benzophenones against UV-Induced Skin Damage. Photochem. Photobiol. Sci..

[166] Dudoit A., Mertz C., Chillet M., Cardinault N., Brat P. (2020). Antifungal Activity of Brazilian Red Propolis Extract and Isolation of Bioactive Fractions by Thin-Layer Chromatography-Bioautography. Food Chem..

[167] Toreti V.C., Sato H.H., Pastore G.M., Park Y.K. (2013). Recent Progress of Propolis for Its Biological and Chemical Compositions and Its Botanical Origin. Evid.-Based Complement. Altern. Med..

[168] Louten J. (2016). Virus Replication. Essential Human Virology.

[169] Kwon M.J., Shin H.M., Perumalsamy H., Wang X., Ahn Y.-J. (2020). Antiviral Effects and Possible Mechanisms of Action of Constituents from Brazilian Propolis and Related Compounds. J. Apic. Res..

[170] González-Búrquez M.D.J., González-Díaz F.R., García-Tovar C.G., Carrillo-Miranda L., Soto-Zárate C.I., Canales-Martínez M.M., Penieres-Carrillo J.G., Crúz-Sánchez T.A., Fonseca-Coronado S. (2018). Comparison between In Vitro Antiviral Effect of Mexican Propolis and Three Commercial Flavonoids against Canine Distemper Virus. Evid.-Based Complement. Altern. Med..

[171] Schnitzler P., Neuner A., Nolkemper S., Zundel C., Nowack H., Sensch K.H., Reichling J. (2010). Antiviral Activity and Mode of Action of Propolis Extracts and Selected Compounds. Phytother. Res..

[172] Labska K., Plodkova H., Pumannova M., Sensch K.H. (2018). Antiviral Activity of Propolis Special Extract GH 2002 against *Varicella zoster* Virus in Vitro. Pharmazie.

[173] Kuropatnicki A.K., Szliszka E., Krol W. (2013). Historical Aspects of Propolis Research in Modern Times. Evid.-Based Complement. Altern. Med..

[174] Yildirim A., Duran G.G., Duran N., Jenedi K., Bolgul B.S., Miraloglu M., Muz M. (2016). Antiviral Activity of Hatay Propolis Against Replication of Herpes Simplex Virus Type 1 and Type 2. Med. Sci. Monit..

[175] Ali A.M., Kunugi H. (2021). Propolis, Bee Honey, and Their Components Protect against Coronavirus Disease 2019 (COVID-19): A Review of In Silico, In Vitro, and Clinical Studies. Molecules.

[176] Refaat H., Naguib Y.W., Elsayed M.M.A., Sarhan H.A.A., Alaaeldin E. (2019). Modified Spraying Technique and Response Surface Methodology for the Preparation and Optimization of Propolis Liposomes of Enhanced Anti-Proliferative Activity against Human Melanoma Cell Line A375. Pharmaceutics.

[177] Refaat H., Mady F.M., Sarhan H.A., Rateb H.S., Alaaeldin E. (2021). Optimization and Evaluation of Propolis Liposomes as a Promising Therapeutic Approach for COVID-19. Int. J. Pharm..

[178] Toutou Z., Fatmi S., Chibani N., Pokajewicz K., Skiba M., Wieczorek P.P., Iguerouada M. (2025). Exploring the Therapeutic Potential of Algerian Propolis: GC/MS Profiling, Protective Inclusion Complex, and In Silico Evaluation Against SARS-COV-2 Main Proteases. Pept. Sci..

[179] Maruta H., He H. (2020). PAK1-Blockers: Potential Therapeutics against COVID-19. Med. Drug Discov..

[180] Amoros M., Simõs C.M.O., Girre L., Sauvager F., Cormier M. (1992). Synergistic Effect of Flavones and Flavonols Against Herpes Simplex Virus Type 1 in Cell Culture. Comparison with the Antiviral Activity of Propolis. J. Nat. Prod..

[181] Mattson M.P., Arumugam T.V. (2018). Hallmarks of Brain Aging: Adaptive and Pathological Modification by Metabolic States. Cell Metab..

[182] Zinger A., Cho W.C., Ben-Yehuda A. (2017). Cancer and Aging—The Inflammatory Connection. Aging Dis..

[183] Rådmark O., Werz O., Steinhilber D., Samuelsson B. (2007). 5-Lipoxygenase: Regulation of Expression and Enzyme Activity. Trends Biochem. Sci..

[184] Silva J.C., Rodrigues S., Feás X., Estevinho L.M. (2012). Antimicrobial Activity, Phenolic Profile and Role in the Inflammation of Propolis. Food Chem. Toxicol..

[185] Viuda-Martos M., Ruiz-Navajas Y., Fernández-López J., Pérez-Alvarez J.A. (2008). Functional Properties of Honey, Propolis, and Royal Jelly. J. Food Sci..

[186] Ikeda R., Yanagisawa M., Takahashi N., Kawada T., Kumazawa S., Yamaotsu N., Nakagome I., Hirono S., Tsuda T. (2011). Brazilian Propolis-Derived Components Inhibit TNF-α-Mediated Downregulation of Adiponectin Expression via Different Mechanisms in 3T3-L1 Adipocytes. Biochim. Biophys. Acta BBA Gen. Subj..

[187] Wang T., Chen L., Wu W., Long Y., Wang R. (2008). Potential Cytoprotection: Antioxidant Defence by Caffeic Acid Phenethyl Ester against Free Radical-Induced Damage of Lipids, DNA, and Proteins. Can. J. Physiol. Pharmacol..

[188] Kumazawa S., Ahn M.-R., Fujimoto T., Kato M. (2010). Radical-Scavenging Activity and Phenolic Constituents of Propolis from Different Regions of Argentina. Nat. Prod. Res..

[189] Song M.-Y., Lee D.-Y., Kim E.-H. (2020). Anti-Inflammatory and Anti-Oxidative Effect of Korean Propolis on Helicobacter Pylori-Induced Gastric Damage in Vitro. J. Microbiol..

[190] Moura S.A., Negri G., Salatino A., Lima L.D., Dourado L.P., Mendes J.B., Andrade S.P., Ferreira M.A., Cara D.C. (2011). Aqueous Extract of Brazilian Green Propolis: Primary Components, Evaluation of Inflammation and Wound Healing by Using Subcutaneous Implanted Sponges. Evid.-Based Complement. Altern. Med..

[191] Du Toit K., Buthelezi S., Bodenstein J. (2010). Anti-Inflammatory and Antibacterial Profiles of Selected Compounds Found in South African Propolis. South Afr. J. Sci..

[192] Jung Y.C., Kim M.E., Yoon J.H., Park P.R., Youn H.-Y., Lee H.-W., Lee J.S. (2014). Anti-Inflammatory Effects of Galangin on Lipopolysaccharide-Activated Macrophages via ERK and NF-κB Pathway Regulation. Immunopharmacol. Immunotoxicol..

[193] Cardenas H., Arango D., Nicholas C., Duarte S., Nuovo G., He W., Voss O., Gonzalez-Mejia M., Guttridge D., Grotewold E. (2016). Dietary Apigenin Exerts Immune-Regulatory Activity in Vivo by Reducing NF-κB Activity, Halting Leukocyte Infiltration and Restoring Normal Metabolic Function. Int. J. Mol. Sci..

[194] Zhang X., Wang G., Gurley E.C., Zhou H. (2014). Flavonoid Apigenin Inhibits Lipopolysaccharide-Induced Inflammatory Response through Multiple Mechanisms in Macrophages. PLoS ONE.

[195] Santos E.O.L., Kabeya L.M., Figueiredo-Rinhel A.S.G., Marchi L.F., Andrade M.F., Piatesi F., Paoliello-Paschoalato A.B., Azzolini A.E.C.S., Lucisano-Valim Y.M. (2014). Flavonols Modulate the Effector Functions of Healthy Individuals’ Immune Complex-Stimulated Neutrophils: A Therapeutic Perspective for Rheumatoid Arthritis. Int. Immunopharmacol..

[196] Batista C.M., Alves A.V.F., Queiroz L.A., Lima B.S., Filho R.N.P., Araújo A.A.S., de Albuquerque Júnior R.L.C., Cardoso J.C. (2018). The Photoprotective and Anti-Inflammatory Activity of Red Propolis Extract in Rats. J. Photochem. Photobiol. B Biol..

[197] Wang Y., Zhu Y., Gao L., Yin H., Xie Z., Wang D., Zhu Z., Han X. (2012). Formononetin Attenuates IL-1β-Induced Apoptosis and NF-κB Activation in INS-1 Cells. Molecules.

[198] Szliszka E., Mertas A., Czuba Z.P., Król W. (2013). Inhibition of Inflammatory Response by Artepillin C in Activated RAW264.7 Macrophages. Evid.-Based Complement. Altern. Med..

[199] Orsatti C.L., Missima F., Pagliarone A.C., Bachiega T.F., Búfalo M.C., Araújo J.P., Sforcin J.M. (2010). Propolis Immunomodulatory Action in Vivo on Toll-like Receptors 2 and 4 Expression and on Pro-inflammatory Cytokines Production in Mice. Phytother. Res..

[200] Balderas-Cordero D., Canales-Alvarez O., Sánchez-Sánchez R., Cabrera-Wrooman A., Canales-Martinez M.M., Rodriguez-Monroy M.A. (2023). Anti-Inflammatory and Histological Analysis of Skin Wound Healing through Topical Application of Mexican Propolis. Int. J. Mol. Sci..

[201] Park E.-H., Kahng J.-H. (1999). Suppressive Effects of Propolis in Rat Adjuvant Arthritis. Arch. Pharmacol. Res..

[202] Nattagh-Eshtivani E., Jokar M., Tabesh H., Nematy M., Safarian M., Pahlavani N., Maddahi M., Khosravi M. (2021). The Effect of Propolis Supplementation on Inflammatory Factors and Oxidative Status in Women with Rheumatoid Arthritis: Design and Research Protocol of a Double-Blind, Randomized Controlled. Contemp. Clin. Trials Commun..

[203] Sawaya A.C.H.F., Barbosa Da Silva Cunha I., Marcucci M.C. (2011). Analytical Methods Applied to Diverse Types of Brazilian Propolis. Chem. Cent. J..

[204] Hossain R., Quispe C., Khan R.A., Saikat A.S.M., Ray P., Ongalbek D., Yeskaliyeva B., Jain D., Smeriglio A., Trombetta D. (2022). Propolis: An Update on Its Chemistry and Pharmacological Applications. Chin. Med..

[205] Tao Y., Wang D., Hu Y., Huang Y., Yu Y., Wang D. (2014). The Immunological Enhancement Activity of Propolis Flavonoids Liposome In Vitro and In Vivo. Evid.-Based Complement. Altern. Med..

[206] Fan Y., Ma L., Zhang W., Xu Y., Zhanxi S.L., Zhi X., Cui E., Song X. (2014). Microemulsion Can Improve the Immune-Enhancing Activity of Propolis Flavonoid on Immunosuppression and Immune Response. Int. J. Biol. Macromol..

[207] Orsi R.O., Funari S.R.C., Soares A.M.V.C., Calvi S.A., Oliveira S.L., Sforcin J.M., Bankova V. (2000). Immunomodulatory Action of Propolis on Macrophage Activation. J. Venom. Anim. Toxins.

[208] Murad J.M., Calvi S.A., Soares A.M.V.C., Bankova V., Sforcin J.M. (2002). Effects of Propolis from Brazil and Bulgaria on Fungicidal Activity of Macrophages against *Paracoccidioides brasiliensis*. J. Ethnopharmacol..

[209] Rebouças-Silva J., Amorim N.A., Jesus-Santos F.H., De Lima J.A., Lima J.B., Berretta A.A., Borges V.M. (2023). Leishmanicidal and Immunomodulatory Properties of Brazilian Green Propolis Extract (EPP-AF^®^) and a Gel Formulation in a Pre-Clinical Model. Front. Pharmacol..

[210] Sforcin J.M., Orsi R.O., Bankova V. (2005). Effect of Propolis, Some Isolated Compounds and Its Source Plant on Antibody Production. J. Ethnopharmacol..

[211] Sforcin J.M. (2007). Propolis and the Immune System: A Review. J. Ethnopharmacol..

[212] Doi K., Fujioka M., Sokuza Y., Ohnishi M., Gi M., Takeshita M., Kumada K., Kakehashi A., Wanibuchi H. (2017). Chemopreventive Action by Ethanol-Extracted Brazilian Green Propolis on Post-Initiation Phase of Inflammation-Associated Rat Colon Tumorigenesis. In Vivo.

[213] Mojarab S., Shahbazzadeh D., Moghbeli M., Eshraghi Y., Bagheri K.P., Rahimi R., Savoji M.A., Mahdavi M. (2020). Immune Responses to HIV-1 Polytope Vaccine Candidate Formulated in Aqueous and Alcoholic Extracts of Propolis: Comparable Immune Responses to Alum and Freund Adjuvants. Microb. Pathog..

[214] Fischer G., Cleff M.B., Dummer L.A., Paulino N., Paulino A.S., Vilela C.D.O., Campos F.S., Storch T., Vargas G.D., Hübner S.D.O. (2007). Adjuvant Effect of Green Propolis on Humoral Immune Response of Bovines Immunized with Bovine Herpesvirus Type 5. Vet. Immunol. Immunopathol..

[215] Fischer G., Conceição F.R., Leite F.P.L., Dummer L.A., Vargas G.D., Hübner S.D.O., Dellagostin O.A., Paulino N., Paulino A.S., Vidor T. (2007). Immunomodulation Produced by a Green Propolis Extract on Humoral and Cellular Responses of Mice Immunized with SuHV-1. Vaccine.

[216] Magnavacca A., Sangiovanni E., Racagni G., Dell’Agli M. (2022). The Antiviral and Immunomodulatory Activities of Propolis: An Update and Future Perspectives for Respiratory Diseases. Med. Res. Rev..

[217] Chinthammit C., Axon D.R., Mollon L., Taylor A.M., Pickering M., Black H., Warholak T., Campbell P.J. (2021). Evaluating the Relationship between Quality Measure Adherence Definitions and Economic Outcomes in Commercial Health Plans: A Retrospective Diabetes Cohort Study. J. Manag. Care Spec. Pharm..

[218] International Diabetes Federation (2021). 2021 IDF Diabetes Atlas.

[219] Deng L., Du C., Song P., Chen T., Rui S., Armstrong D.G., Deng W. (2021). The Role of Oxidative Stress and Antioxidants in Diabetic Wound Healing. Oxidative Med. Cell. Longev..

[220] Luc K., Schramm-Luc A., Guzik T.J., Mikolajczyk T.P. (2019). Oxidative Stress and Inflammatory Markers in Prediabetes and Diabetes. J. Physiol. Pharmacol..

[221] Chiasson J.-L., Josse R.G., Gomis R., Hanefeld M., Karasik A., Laakso M. (2002). Acarbose for Prevention of Type 2 Diabetes Mellitus: The STOP-NIDDM Randomised Trial. Lancet.

[222] Chiba S. (1997). Molecular Mechanism in *α*-Glucosidase and Glucoamylase. Biosci. Biotechnol. Biochem..

[223] El Adaouia Taleb R., Djebli N., Chenini H., Sahin H., Kolayli S. (2020). In Vivo and in Vitro Anti-diabetic Activity of Ethanolic Propolis Extract. J. Food Biochem..

[224] Popova M., Lyoussi B., Aazza S., Antunes D., Bankova V., Miguel G. (2015). Antioxidant and α-Glucosidase Inhibitory Properties and Chemical Profiles of Moroccan Propolis. Nat. Prod. Commun..

[225] Hernández-Martínez J.A., Zepeda-Bastida A., Morales-Rodríguez I., Fernández-Luqueño F., Campos-Montiel R., Hereira-Pacheco S.E., Medina-Pérez G. (2024). Potential Antidiabetic Activity of Apis Mellifera Propolis Extraction Obtained with Ultrasound. Foods.

[226] Alaribe C.S., Esposito T., Sansone F., Sunday A., Pagano I., Piccinelli A.L., Celano R., Cuesta Rubio O., Coker H.A., Nabavi S.M. (2021). Nigerian Propolis: Chemical Composition, Antioxidant Activity and α-Amylase and α-Glucosidase Inhibition. Nat. Prod. Res..

[227] El-Guendouz S., Aazza S., Lyoussi B., Antunes M.D., Faleiro M.L., Miguel M.G. (2016). Anti-acetylcholinesterase, Antidiabetic, Anti-inflammatory, Antityrosinase and Antixanthine Oxidase Activities of Moroccan Propolis. Int. J. Food Sci. Technol..

[228] Vongsak B., Kongkiatpaiboon S., Jaisamut S., Machana S., Pattarapanich C. (2015). In Vitro Alpha Glucosidase Inhibition and Free-Radical Scavenging Activity of Propolis from Thai Stingless Bees in Mangosteen Orchard. Rev. Bras. Farm..

[229] Zhu W., Li Y.-H., Chen M.-L., Hu F.-L. (2011). Protective Effects of Chinese and Brazilian Propolis Treatment against Hepatorenal Lesion in Diabetic Rats. Hum. Exp. Toxicol..

[230] Chen L.-H., Chien Y.-W., Chang M.-L., Hou C.-C., Chan C.-H., Tang H.-W., Huang H.-Y. (2018). Taiwanese Green Propolis Ethanol Extract Delays the Progression of Type 2 Diabetes Mellitus in Rats Treated with Streptozotocin/High-Fat Diet. Nutrients.

[231] El Menyiy N., Al-Waili N., El Ghouizi A., El-Guendouz S., Salom K., Lyoussi B. (2019). Potential Therapeutic Effect of Moroccan Propolis in Hyperglycemia, Dyslipidemia, and Hepatorenal Dysfunction in Diabetic Rats. Iran. J. Basic Med. Sci..

[232] Al-Hariri M.T., Eldin T.A.G., Al-Harb M.M. (2016). Protective Effect and Potential Mechanisms of Propolis on Streptozotocin-Induced Diabetic Rats. J. Taibah Univ. Med. Sci..

[233] Laaroussi H., Bakour M., Ousaaid D., Aboulghazi A., Ferreira-Santos P., Genisheva Z., Teixeira J.A., Lyoussi B. (2020). Effect of Antioxidant-Rich Propolis and Bee Pollen Extracts against D-Glucose Induced Type 2 Diabetes in Rats. Food Res. Int..

[234] Samadi N., Mozaffari-Khosravi H., Rahmanian M., Askarishahi M. (2017). Effects of bee propolis supplementation on glycemic control, lipid profile and insulin resistance indices in patients with type 2 diabetes: A randomized, double-blind clinical trial. J. Integr. Med..

[235] Sartori D., Kawakami C., Orsatti C., Sforcin J. (2009). Propolis Effect on Streptozotocin-Induced Diabetic Rats. J. Venom. Anim. Toxins Incl. Trop. Dis..

[236] Aoi W., Hosogi S., Niisato N., Yokoyama N., Hayata H., Miyazaki H., Kusuzaki K., Fukuda T., Fukui M., Nakamura N. (2013). Improvement of Insulin Resistance, Blood Pressure and Interstitial pH in Early Developmental Stage of Insulin Resistance in OLETF Rats by Intake of Propolis Extracts. Biochem. Biophys. Res. Commun..

[237] Babatunde I.R., Abdulbasit A., Oladayo M.I., Olasile O.I., Olamide F.R., Gbolahan B.W. (2015). Hepatoprotective and Pancreatoprotective Properties of the Ethanolic Extract of Nigerian Propolis. J. Intercult. Ethnopharmacol..

[238] Mustafa I. (2016). Nigerian Propolis Improves Blood Glucose, Glycated Hemoglobin (HbA1c), VLDL and HDL Levels in Rat Models of Diabetes. J. Intercult. Ethnopharmacol..

[239] Ochoa-Morales P.D., González-Ortiz M., Martínez-Abundis E., Pérez-Rubio K.G., Patiño-Laguna A.D.J. (2023). Anti-Hyperglycemic Effects of Propolis or Metformin in Type 2 Diabetes Mellitus: A Randomized Controlled Trial. Int. J. Vitam. Nutr. Res..

[240] Medellín-Luna M.F., Castañeda-Delgado J.E., Martínez-Balderas V.Y., Cervantes-Villagrana A.R. (2019). Medicinal Plant Extracts and Their Use as Wound Closure Inducing Agents. J. Med. Food.

[241] Yang J., Pi A., Yan L., Li J., Nan S., Zhang J., Hao Y. (2022). Research Progress on Therapeutic Effect and Mechanism of Propolis on Wound Healing. Evid.-Based Complement. Altern. Med..

[242] Gurtner G.C., Werner S., Barrandon Y., Longaker M.T. (2008). Wound Repair and Regeneration. Nature.

[243] Rojczyk E., Klama-Baryła A., Łabuś W., Wilemska-Kucharzewska K., Kucharzewski M. (2020). Historical and Modern Research on Propolis and Its Application in Wound Healing and Other Fields of Medicine and Contributions by Polish Studies. J. Ethnopharmacol..

[244] Baum C.L., Arpey C.J. (2006). Normal Cutaneous Wound Healing: Clinical Correlation with Cellular and Molecular Events. Dermatol. Surg..

[245] Hiromatsu Y., Toda S. (2003). Mast Cells and Angiogenesis. Microsc. Res. Tech..

[246] Walsh L.J. (2003). Mast cells and oral inflammation. Crit. Rev. Oral Biol. Med..

[247] Noli C., Miolo A. (2001). The Mast Cell in Wound Healing. Vet. Dermatol..

[248] Nishida K., Hasegawa A., Yamasaki S., Uchida R., Ohashi W., Kurashima Y., Kunisawa J., Kimura S., Iwanaga T., Watarai H. (2019). Mast Cells Play Role in Wound Healing through the ZnT2/GPR39/IL-6 Axis. Sci. Rep..

[249] Weller K., Foitzik K., Paus R., Syska W., Maurer M., Weller K., Foitzik K., Paus R., Syska W., Maurer M. (2006). Mast Cells Are Required for Normal Healing of Skin Wounds in Mice. FASEB J..

[250] El-Sakhawy M., Salama A., Tohamy H.-A.S. (2023). Applications of Propolis-Based Materials in Wound Healing. Arch. Dermatol. Res..

[251] Barroso P.R., Lopes-Rocha R., Pereira E.M.F., Marinho S.A., De Miranda J.L., Lima N.L., Verli F.D. (2012). Effect of Propolis on Mast Cells in Wound Healing. Inflammopharmacology.

[252] Oryan A., Alemzadeh E., Moshiri A. (2018). Potential Role of Propolis in Wound Healing: Biological Properties and Therapeutic Activities. Biomed. Pharmacother..

[253] Stojko M., Wolny D., Włodarczyk J. (2021). Nonwoven Releasing Propolis as a Potential New Wound Healing Method—A Review. Molecules.

[254] Ramadan A., Soliman G., Mahmoud S.S., Nofal S.M., Abdel-Rahman R.F. (2012). Evaluation of the Safety and Antioxidant Activities of Crocus Sativus and Propolis Ethanolic Extracts. J. Saudi Chem. Soc..

[255] Ramos I.F.D.A.S., Biz M.T., Paulino N., Scremin A., Della Bona Á., Barletta F.B., Figueiredo J.A.P.D. (2012). Histopathological Analysis of Corticosteroid-Antibiotic Preparation and Propolis Paste Formulation as Intracanal Medication after Pulpectomy: An in Vivo Study. J. Appl. Oral Sci..

[256] Martinotti S., Pellavio G., Laforenza U., Ranzato E. (2019). Propolis Induces AQP3 Expression: A Possible Way of Action in Wound Healing. Molecules.

[257] Inui S., Hosoya T., Shimamura Y., Masuda S., Ogawa T., Kobayashi H., Shirafuji K., Moli R.T., Kozone I., Shin-ya K. (2012). Solophenols B–D and Solomonin: New Prenylated Polyphenols Isolated from Propolis Collected from The Solomon Islands and Their Antibacterial Activity. J. Agric. Food Chem..

[258] Ayad A.S., Hébert M.P.A., Doiron J.A., Loucif-Ayad W., Daas T., Smagghe G., Alburaki M., Barnett D.A., Touaibia M., Surette M.E. (2024). Algerian Propolis from Distinct Geographical Locations: Chemical Profiles, Antioxidant Capacity, Cytotoxicity and Inhibition of 5-Lipoxygenase Product Biosynthesis. Chem. Biodivers..

[259] Búfalo M.C., Ferreira I., Costa G., Francisco V., Liberal J., Cruz M.T., Lopes M.C., Batista M.T., Sforcin J.M. (2013). Propolis and Its Constituent Caffeic Acid Suppress LPS-Stimulated pro-Inflammatory Response by Blocking NF-κB and MAPK Activation in Macrophages. J. Ethnopharmacol..

[260] Franchin M., Colón D.F., Da Cunha M.G., Castanheira F.V.S., Saraiva A.L.L., Bueno-Silva B., Alencar S.M., Cunha T.M., Rosalen P.L. (2016). Neovestitol, an Isoflavonoid Isolated from Brazilian Red Propolis, Reduces Acute and Chronic Inflammation: Involvement of Nitric Oxide and IL-6. Sci. Rep..

[261] Hozzein W.N., Badr G., Al Ghamdi A.A., Sayed A., Al-Waili N.S., Garraud O. (2015). Topical Application of Propolis Enhances Cutaneous Wound Healing by Promoting TGF-Beta/Smad-Mediated Collagen Production in a Streptozotocin-Induced Type I Diabetic Mouse Model. Cell. Physiol. Biochem..

[262] Cao X.-P., Chen Y.-F., Zhang J.-L., You M.-M., Wang K., Hu F.-L. (2017). Mechanisms Underlying the Wound Healing Potential of Propolis Based on Its in Vitro Antioxidant Activity. Phytomedicine.

[263] Kawai M., Hirano T., Higa S., Arimitsu J., Maruta M., Kuwahara Y., Ohkawara T., Hagihara K., Yamadori T., Shima Y. (2007). Flavonoids and Related Compounds as Anti-Allergic Substances. Allergol. Int..

[264] Yano S., Umeda D., Yamashita T., Ninomiya Y., Sumida M., Fujimura Y., Yamada K., Tachibana H. (2007). Dietary Flavones Suppresses IgE and Th2 Cytokines in OVA-Immunized BALB/c Mice. Eur. J. Nutr..

[265] Nakamura R., Nakamura R., Watanabe K., Oka K., Ohta S., Mishima S., Teshima R. (2010). Effects of Propolis from Different Areas on Mast Cell Degranulation and Identification of the Effective Components in Propolis. Int. Immunopharmacol..

[266] Bae Y., Lee S., Kim S.-H. (2011). Chrysin Suppresses Mast Cell-Mediated Allergic Inflammation: Involvement of Calcium, Caspase-1 and Nuclear Factor-κB. Toxicol. Appl. Pharmacol..

[267] Cho M.S., Park W.S., Jung W.-K., Qian Z., Lee D.-S., Choi J.-S., Lee D.-Y., Park S.-G., Seo S.-K., Kim H.-J. (2014). Caffeic Acid Phenethyl Ester Promotes Anti-Inflammatory Effects by Inhibiting MAPK and NF-κB Signaling in Activated HMC-1 Human Mast Cells. Pharm. Biol..

[268] Nader M.A. (2013). Caffeic Acid Phenethyl Ester Attenuates IgE-Induced Immediate Allergic Reaction. Inflammopharmacology.

[269] Kapare H.S., Giram P.S., Raut S.S., Gaikwad H.K., Paiva-Santos A.C. (2023). Formulation Development and Evaluation of Indian Propolis Hydrogel for Wound Healing. Gels.

[270] Koo H., Rosalen P.L., Cury J.A., Park Y.K., Bowen W.H. (2002). Effects of Compounds Found in Propolis on *Streptococcus Mutans* Growth and on Glucosyltransferase Activity. Antimicrob. Agents Chemother..

[271] Conceição M., Gushiken L.F.S., Aldana-Mejía J.A., Tanimoto M.H., Ferreira M.V.D.S., Alves A.C.M., Miyashita M.N., Bastos J.K., Beserra F.P., Pellizzon C.H. (2022). Histological, Immunohistochemical and Antioxidant Analysis of Skin Wound Healing Influenced by the Topical Application of Brazilian Red Propolis. Antioxidants.

[272] Abdellatif M.M., Elakkad Y.E., Elwakeel A.A., Allam R.M., Mousa M.R. (2021). Formulation and Characterization of Propolis and Tea Tree Oil Nanoemulsion Loaded with Clindamycin Hydrochloride for Wound Healing: In-Vitro and in-Vivo Wound Healing Assessment. Saudi Pharm. J..

[273] Chirumbolo S. (2012). Flavonoids in Propolis Acting on Mast Cell-Mediated Wound Healing. Inflammopharmacology.

[274] Canales-Alvarez O., Canales-Martinez M.M., Dominguez-Verano P., Balderas-Cordero D., Madrigal-Bujaidar E., Álvarez-González I., Rodriguez-Monroy M.A. (2024). Effect of Mexican Propolis on Wound Healing in a Murine Model of Diabetes Mellitus. Int. J. Mol. Sci..

[275] Eyarefe O.D., Ozota C.A., Jarikre T.A., Emikpe B.O. (2019). Pathological and Immunohistochemical Evaluation of Wound Healing Potential of Nigerian Bee Propolis in Albino Rats. Comp. Clin. Pathol..

[276] Moghtaday Khorasgani E., Karimi A.H., Nazem M.R. (2010). A Comparison of Healing Effects of Propolis and Silver Sulfadiazine on Full Thickness SkinWounds in Rats. Pak. Vet. J..

[277] Kucharzewski M., Kózka M., Urbanek T. (2013). Topical Treatment of Nonhealing Venous Leg Ulcer with Propolis Ointment. Evid.-Based Complement. Altern. Med..

[278] Samet N., Laurent C., Susarla S.M., Samet-Rubinsteen N. (2007). The Effect of Bee Propolis on Recurrent Aphthous Stomatitis: A Pilot Study. Clin. Oral Investig..

[279] Olczyk P., Wisowski G., Komosinska-Vassev K., Stojko J., Klimek K., Olczyk M., Kozma E.M. (2013). Propolis Modifies Collagen Types I and III Accumulation in the Matrix of Burnt Tissue. Evid.-Based Complement. Altern. Med..

[280] Staniczek J., Jastrzębska-Stojko Ż., Stojko R. (2021). Biological Activity of Propolis Ointment with the Addition of 1% Nanosilver in the Treatment of Experimentally-Evoked Burn Wounds. Polymers.

[281] Afkhamizadeh M., Aboutorabi R., Ravari H., Fathi Najafi M., Ataei Azimi S., Javadian Langaroodi A., Yaghoubi M.A., Sahebkar A. (2018). Topical Propolis Improves Wound Healing in Patients with Diabetic Foot Ulcer: A Randomized Controlled Trial. Nat. Prod. Res..

[282] Mujica V., Orrego R., Fuentealba R., Leiva E., Zúñiga-Hernández J. (2019). Propolis as an Adjuvant in the Healing of Human Diabetic Foot Wounds Receiving Care in the Diagnostic and Treatment Centre from the Regional Hospital of Talca. J. Diabetes Res..

[283] Da Rosa C., Bueno I.L., Quaresma A.C.M., Longato G.B. (2022). Healing Potential of Propolis in Skin Wounds Evidenced by Clinical Studies. Pharmaceuticals.

[284] Russo A., Longo R., Vanella A. (2002). Antioxidant Activity of Propolis: Role of Caffeic Acid Phenethyl Ester and Galangin. Fitoterapia.

[285] Kumazawa S., Hamasaka T., Nakayama T. (2004). Antioxidant Activity of Propolis of Various Geographic Origins. Food Chem..

[286] Valente M.J., Baltazar A.F., Henrique R., Estevinho L., Carvalho M. (2011). Biological Activities of Portuguese Propolis: Protection against Free Radical-Induced Erythrocyte Damage and Inhibition of Human Renal Cancer Cell Growth in Vitro. Food Chem. Toxicol..

[287] Okińczyc P., Widelski J., Szperlik J., Żuk M., Mroczek T., Skalicka-Woźniak K., Sakipova Z., Widelska G., Kuś P.M. (2021). Impact of Plant Origin on Eurasian Propolis on Phenolic Profile and Classical Antioxidant Activity. Biomolecules.

[288] Olczyk P., Komosińska-Vassev K., Winsz-Szczotka K., Koźma E.M., Wisowski G., Stojko J., Klimek K., Olczyk K. (2012). Propolis Modulates Vitronectin, Laminin, and Heparan Sulfate/Heparin Expression during Experimental Burn Healing. J. Zhejiang Univ. Sci. B.

[289] Gregory S.R., Piccolo N., Piccolo M.T., Piccolo M.S., Heggers J.P. (2002). Comparison of Propolis Skin Cream to Silver Sulfadiazine: A Naturopathic Alternative to Antibiotics in Treatment of Minor Burns. J. Altern. Complement. Med..

[290] Olczyk P., Ramos P., Bernas M., Komosinska-Vassev K., Stojko J., Pilawa B. (2013). Application of Electron Paramagnetic Resonance Spectroscopy to Comparative Examination of Different Groups of Free Radicals in Thermal Injuries Treated with Propolis and Silver Sulphadiazine. Evid.-Based Complement. Altern. Med..

[291] Pessolato A.G.T., Martins D.D.S., Ambrósio C.E., Mançanares C.A.F., De Carvalho A.F. (2011). Propolis and Amnion Reepithelialise Second-Degree Burns in Rats. Burns.

[292] Siegel R.L., Miller K.D., Fuchs H.E., Jemal A. (2021). Cancer Statistics, 2021. CA Cancer J. Clin..

[293] Dolgin E. (2018). Bringing down the Cost of Cancer Treatment. Nature.

[294] Vasan N., Baselga J., Hyman D.M. (2019). A View on Drug Resistance in Cancer. Nature.

[295] Darvishi N., Yousefinejad V., Akbari M.E., Abdi M., Moradi N., Darvishi S., Mehrabi Y., Ghaderi E., Jamshidi-Naaeini Y., Ghaderi B. (2020). Antioxidant and Anti-Inflammatory Effects of Oral Propolis in Patients with Breast Cancer Treated with Chemotherapy: A Randomized Controlled Trial. J. Herb. Med..

[296] Ebeid S.A., Abd El Moneim N.A., El-Benhawy S.A., Hussain N.G., Hussain M.I. (2016). Assessment of the Radioprotective Effect of Propolis in Breast Cancer Patients Undergoing Radiotherapy. New Perspective for an Old Honeybee Product. J. Radiat. Res. Appl. Sci..

[297] Watanabe M.A.E., Amarante M.K., Conti B.J., Sforcin J.M. (2011). Cytotoxic Constituents of Propolis Inducing Anticancer Effects: A Review. J. Pharm. Pharmacol..

[298] Atanasov A.G., Zotchev S.B., Dirsch V.M., The International Natural Product Sciences Taskforce (2021). Natural Products in Drug Discovery: Advances and Opportunities. Nat. Rev. Drug Discov..

[299] Sun G., Qiu Z., Wang W., Sui X., Sui D. (2016). Flavonoids Extraction from Propolis Attenuates Pathological Cardiac Hypertrophy through PI3K/AKT Signaling Pathway. Evid.-Based Complement. Altern. Med..

[300] Fuliang H., Hepburn H., Xuan H., Chen M., Daya S., Radloff S. (2005). Effects of Propolis on Blood Glucose, Blood Lipid and Free Radicals in Rats with Diabetes Mellitus. Pharmacol. Res..

[301] Ertürküner S.P., Yaprak Saraç E., Göçmez S.S., Ekmekçi H., Öztürk Z.B., Seçkin İ., Sever Ö., Keskinbora K. (2015). Anti-Inflammatory and Ultrastructural Effects of Turkish Propolis in a Rat Model of Endotoxin-Induced Uveitis. Folia Histochem. Cytobiol..

[302] Demir S., Aliyazicioglu Y., Turan I., Misir S., Mentese A., Yaman S.O., Akbulut K., Kilinc K., Deger O. (2016). Antiproliferative and Proapoptotic Activity of Turkish Propolis on Human Lung Cancer Cell Line. Nutr. Cancer.

[303] Spilioti E., Jaakkola M., Tolonen T., Lipponen M., Virtanen V., Chinou I., Kassi E., Karabournioti S., Moutsatsou P. (2014). Phenolic Acid Composition, Antiatherogenic and Anticancer Potential of Honeys Derived from Various Regions in Greece. PLoS ONE.

[304] Valença I., Morais-Santos F., Miranda-Gonçalves V., Ferreira A.M., Almeida-Aguiar C., Baltazar F. (2013). Portuguese Propolis Disturbs Glycolytic Metabolism of Human Colorectal Cancer in Vitro. BMC Complement. Altern. Med..

[305] Xuan H., Li Z., Yan H., Sang Q., Wang K., He Q., Wang Y., Hu F. (2014). Antitumor Activity of Chinese Propolis in Human Breast Cancer MCF-7 and MDA-MB-231 Cells. Evid.-Based Complement. Altern. Med..

[306] Rzepecka-Stojko A., Kabała-Dzik A., Moździerz A., Kubina R., Wojtyczka R., Stojko R., Dziedzic A., Jastrzębska-Stojko Ż., Jurzak M., Buszman E. (2015). Caffeic Acid Phenethyl Ester and Ethanol Extract of Propolis Induce the Complementary Cytotoxic Effect on Triple-Negative Breast Cancer Cell Lines. Molecules.

[307] Rivera-Yañez N., Rodriguez-Canales M., Nieto-Yañez O., Jimenez-Estrada M., Ibarra-Barajas M., Canales-Martinez M.M., Rodriguez-Monroy M.A. (2018). Hypoglycaemic and Antioxidant Effects of Propolis of Chihuahua in a Model of Experimental Diabetes. Evid.-Based Complement. Altern. Med..

[308] Banskota A.H., Tezuka Y., Adnyana I.K., Midorikawa K., Matsushige K., Message D., Huertas A.A.G., Kadota S. (2000). Cytotoxic, Hepatoprotective and Free Radical Scavenging Effects of Propolis from Brazil, Peru, the Netherlands and China. J. Ethnopharmacol..

[309] Búfalo M.C., Candeias J.M.G., Sousa J.P.B., Bastos J.K., Sforcin J.M. (2010). In Vitro Cytotoxic Activity of *Baccharis Dracunculifolia* and Propolis against HEp-2 Cells. Nat. Prod. Res..

[310] Mora D.P.P., Santiago K.B., Conti B.J., De Oliveira Cardoso E., Conte F.L., Oliveira L.P.G., De Assis Golim M., Uribe J.F.C., Gutiérrez R.M., Buitrago M.R. (2019). The Chemical Composition and Events Related to the Cytotoxic Effects of Propolis on Osteosarcoma Cells: A Comparative Assessment of Colombian Samples. Phytother. Res..

[311] Adan A., Kiraz Y., Baran Y. (2016). Cell Proliferation and Cytotoxicity Assays. Curr. Pharm. Biotechnol..

[312] Ishihara M., Naoi K., Hashita M., Itoh Y., Suzui M. (2009). Growth Inhibitory Activity of Ethanol Extracts of Chinese and Brazilian Propolis in Four Human Colon Carcinoma Cell Lines. Oncol. Rep..

[313] Misir S., Aliyazicioglu Y., Demir S., Turan I., Hepokur C. (2020). Effect of Turkish Propolis on miRNA Expression, Cell Cycle, and Apoptosis in Human Breast Cancer (MCF-7) Cells. Nutr. Cancer.

[314] Al-Oudat B.A., Alqudah M.A., Audat S.A., Al-Balas Q.A., El-Elimat T., Hassan M.A., Frhat I.N., Azaizeh M.M. (2019). Design, Synthesis, and Biologic Evaluation of Novel Chrysin Derivatives as Cytotoxic Agents and Caspase-3/7 Activators. Drug Des. Dev. Ther..

[315] Zingue S., Maxeiner S., Rutz J., Ndinteh D.T., Chun F.K.-H., Fohouo F.T., Njamen D., Blaheta R.A. (2020). Ethanol-extracted Cameroonian Propolis: Antiproliferative Effects and Potential Mechanism of Action in Prostate Cancer. Andrologia.

[316] KabaÅ‚a-Dzik A., Rzepecka-Stojko A., Kubina R., Iriti M., Wojtyczka R.D., Buszman E., Stojko J. (2018). Flavonoids, Bioactive Components of Propolis, Exhibit Cytotoxic Activity and Induce Cell Cycle Arrest and Apoptosis in Human Breast Cancer Cells MDA-MB-231 and MCF-7–a Comparative Study. Cell. Mol. Biol..

[317] Wang W., Heideman L., Chung C.S., Pelling J.C., Koehler K.J., Birt D.F. (2000). Cell-Cycle Arrest at G2/M and Growth Inhibition by Apigenin in Human Colon Carcinoma Cell Lines. Mol. Carcinog..

[318] Shahinozzaman M., Basak B., Emran R., Rozario P., Obanda D.N. (2020). Artepillin C: A Comprehensive Review of Its Chemistry, Bioavailability, and Pharmacological Properties. Fitoterapia.

[319] Coskun Z.M., Ersoz M., Gecili M., Ozden A., Acar A. (2020). Cytotoxic and Apoptotic Effects of Ethanolic Propolis Extract on C6 Glioma Cells. Environ. Toxicol..

[320] Scifo C., Cardile V., Russo A., Consoli R., Vancheri C., Capasso F., Vanella A., Renis M. (2004). Resveratrol and Propolis as Necrosis or Apoptosis Inducers in Human Prostate Carcinoma Cells. Oncol. Res..

[321] Oliveira L.P.G., Conte F.L., Cardoso E.D.O., Conti B.J., Santiago K.B., Golim M.D.A., Cruz M.T., Sforcin J.M. (2016). Immunomodulatory/Inflammatory Effects of Geopropolis Produced by *Melipona fasciculata* Smith in Combination with Doxorubicin on THP-1 Cells. J. Pharm. Pharmacol..

[322] Wadhwa R., Nigam N., Bhargava P., Dhanjal J.K., Goyal S., Grover A., Sundar D., Ishida Y., Terao K., Kaul S.C. (2016). Molecular Characterization and Enhancement of Anticancer Activity of Caffeic Acid Phenethyl Ester by γ Cyclodextrin. J. Cancer.

[323] Frión-Herrera Y., Gabbia D., Scaffidi M., Zagni L., Cuesta-Rubio O., De Martin S., Carrara M. (2020). Cuban Brown Propolis Interferes in the Crosstalk between Colorectal Cancer Cells and M2 Macrophages. Nutrients.

[324] Ishida Y., Gao R., Shah N., Bhargava P., Furune T., Kaul S.C., Terao K., Wadhwa R. (2018). Anticancer Activity in Honeybee Propolis: Functional Insights to the Role of Caffeic Acid Phenethyl Ester and Its Complex With γ-Cyclodextrin. Integr. Cancer Ther..

[325] Banzato T.P., Gubiani J.R., Bernardi D.I., Nogueira C.R., Monteiro A.F., Juliano F.F., De Alencar S.M., Pilli R.A., Lima C.A.D., Longato G.B. (2020). Antiproliferative Flavanoid Dimers Isolated from Brazilian Red Propolis. J. Nat. Prod..

[326] De Carvalho F.M.D.A., Schneider J.K., De Jesus C.V.F., De Andrade L.N., Amaral R.G., David J.M., Krause L.C., Severino P., Soares C.M.F., Caramão Bastos E. (2020). Brazilian Red Propolis: Extracts Production, Physicochemical Characterization, and Cytotoxicity Profile for Antitumor Activity. Biomolecules.

[327] Kamiya T., Nishihara H., Hara H., Adachi T. (2012). Ethanol Extract of Brazilian Red Propolis Induces Apoptosis in Human Breast Cancer MCF-7 Cells through Endoplasmic Reticulum Stress. J. Agric. Food Chem..

[328] Kohtz P.D., Halpern A.L., Eldeiry M.A., Hazel K., Kalatardi S., Ao L., Meng X., Reece T.B., Fullerton D.A., Weyant M.J. (2019). Toll-Like Receptor-4 Is a Mediator of Proliferation in Esophageal Adenocarcinoma. Ann. Thorac. Surg..

[329] Jiang X., Xie H., Li C., You M., Zheng Y., Li G.Q., Chen X., Zhang C., Hu F. (2020). Chinese Propolis Inhibits the Proliferation of Human Gastric Cancer Cells by Inducing Apoptosis and Cell Cycle Arrest. Evid.-Based Complement. Altern. Med..

[330] Sepúlveda C., Núñez O., Torres A., Guzmán L., Wehinger S. (2020). Antitumor Activity of Propolis: Recent Advances in Cellular Perspectives, Animal Models and Possible Applications. Food Rev. Int..

[331] Bailon-Moscoso N., Cevallos-Solorzano G., Romero-Benavides J., Ramirez Orellana M. (2017). Natural Compounds as Modulators of Cell Cycle Arrest: Application for Anticancer Chemotherapies. Curr. Genom..

[332] Kashani B., Zandi Z., Pourbagheri-Sigaroodi A., Bashash D., Ghaffari S.H. (2021). The Role of Toll-like Receptor 4 (TLR4) in Cancer Progression: A Possible Therapeutic Target?. J. Cell. Physiol..

[333] Kimoto T., Aga M., Hino K., Koya-Miyata S., Yamamoto Y., Micallef M.J., Hanaya T., Arai S., Ikeda M., Kurimoto M. (2001). Apoptosis of Human Leukemia Cells Induced by Artepillin C, an Active Ingredient of Brazilian Propolis. Anticancer Res..

[334] Pai J.-T., Lee Y.-C., Chen S.-Y., Leu Y.-L., Weng M.-S. (2018). Propolin C Inhibited Migration and Invasion via Suppression of EGFR-Mediated Epithelial-to-Mesenchymal Transition in Human Lung Cancer Cells. Evid.-Based Complement. Altern. Med..

[335] Chiang K.-C., Yang S.-W., Chang K.-P., Feng T.-H., Chang K.-S., Tsui K.-H., Shin Y.-S., Chen C.-C., Chao M., Juang H.-H. (2018). Caffeic Acid Phenethyl Ester Induces N-Myc Downstream Regulated Gene 1 to Inhibit Cell Proliferation and Invasion of Human Nasopharyngeal Cancer Cells. Int. J. Mol. Sci..

[336] Frión-Herrera Y., Díaz-García A., Ruiz-Fuentes J., Rodríguez-Sánchez H., Maurício Sforcin J. (2018). Mechanisms Underlying the Cytotoxic Effect of Propolis on Human Laryngeal Epidermoid Carcinoma Cells. Nat. Prod. Res..

[337] Boik J. (2001). Natural Compounds in Cancer Therapy: Promising Nontoxic Antitumor Agents from Plants and Other Natural Sources.

[338] Ganesan M., Kanimozhi G., Pradhapsingh B., Khan H.A., Alhomida A.S., Ekhzaimy A., Brindha G., Prasad N.R. (2021). Phytochemicals Reverse P-Glycoprotein Mediated Multidrug Resistance via Signal Transduction Pathways. Biomed. Pharmacother..

[339] Mansoori B., Mohammadi A., Davudian S., Shirjang S., Baradaran B. (2017). The Different Mechanisms of Cancer Drug Resistance: A Brief Review. Adv. Pharm. Bull..

[340] Kebsa W., Lahouel M., Rouibah H., Zihlif M., Ahram M., Abu-Irmaileh B., Mustafa E., Al-Ameer H.J., Al Shhab M. (2018). Reversing Multidrug Resistance in Chemo-Resistant Human Lung Adenocarcinoma (A549/DOX) Cells by Algerian Propolis Through Direct Inhibiting the P-Gp Efflux-Pump, G0/G1 Cell Cycle Arrest and Apoptosis Induction. Anti-Cancer Agents Med. Chem..

[341] Abubakar M.B., Abdullah W.Z., Sulaiman S.A., Ang B.S. (2014). Polyphenols as Key Players for the Antileukaemic Effects of Propolis. Evid.-Based Complement. Altern. Med..

[342] Bogdanov S. (2016). Propolis: Composition, Health, Medicine: A Review.

[343] Kabała-Dzik A., Rzepecka-Stojko A., Kubina R., Jastrzębska-Stojko Ż., Stojko R., Wojtyczka R., Stojko J. (2017). Comparison of Two Components of Propolis: Caffeic Acid (CA) and Caffeic Acid Phenethyl Ester (CAPE) Induce Apoptosis and Cell Cycle Arrest of Breast Cancer Cells MDA-MB-231. Molecules.

[344] Xiang D., Wang D., He Y., Xie J., Zhong Z., Li Z., Xie J. (2006). Caffeic Acid Phenethyl Ester Induces Growth Arrest and Apoptosis of Colon Cancer Cells via the β-Catenin/T-Cell Factor Signaling. Anti-Cancer Drugs.

[345] Ren X., Liu J., Hu L., Liu Q., Wang D., Ning X. (2019). Caffeic Acid Phenethyl Ester Inhibits the Proliferation of HEp2 Cells by Regulating Stat3/Plk1 Pathway and Inducing S Phase Arrest. Biol. Pharm. Bull..

[346] Chang H., Wang Y., Yin X., Liu X., Xuan H. (2017). Ethanol Extract of Propolis and Its Constituent Caffeic Acid Phenethyl Ester Inhibit Breast Cancer Cells Proliferation in Inflammatory Microenvironment by Inhibiting TLR4 Signal Pathway and Inducing Apoptosis and Autophagy. BMC Complement. Altern. Med..

[347] Banskota A.H., Nagaoka T., Sumioka L.Y., Tezuka Y., Awale S., Midorikawa K., Matsushige K., Kadota S. (2002). Antiproliferative Activity of the Netherlands Propolis and Its Active Principles in Cancer Cell Lines. J. Ethnopharmacol..

[348] Goncalves P., Araujo J., Pinho M.J., Martel F. (2011). In Vitro Studies on the Inhibition of Colon Cancer by Butyrate and Polyphenolic Compounds. Nutr. Cancer.

[349] Naz S., Imran M., Rauf A., Orhan I.E., Shariati M.A., Iahtisham U.-H., Iqra Y., Shahbaz M., Qaisrani T.B., Shah Z.A. (2019). Chrysin: Pharmacological and Therapeutic Properties. Life Sci..

[350] Noureddine H., Hage-Sleiman R., Wehbi B., Fayyad-Kazan H., Hayar S., Traboulssi M., Alyamani O.A., Faour W.H., ElMakhour Y. (2017). Chemical Characterization and Cytotoxic Activity Evaluation of Lebanese Propolis. Biomed. Pharmacother..

[351] Benguedouar L., Lahouel M., Gangloff S.C., Durlach A., Grange F., Bernard P., Antonicelli F. (2016). Ethanolic Extract of Algerian Propolis and Galangin Decreased Murine Melanoma Tumor Progression in Mice. Anti-Cancer Agents Med. Chem..

[352] Liu C., Ma M., Zhang J., Gui S., Zhang X., Xue S. (2017). Galangin Inhibits Human Osteosarcoma Cells Growth by Inducing Transforming Growth Factor-Β1-Dependent Osteogenic Differentiation. Biomed. Pharmacother..

[353] Liu D., You P., Luo Y., Yang M., Liu Y. (2018). Galangin Induces Apoptosis in MCF-7 Human Breast Cancer Cells Through Mitochondrial Pathway and Phosphatidylinositol 3-Kinase/Akt Inhibition. Pharmacology.

[354] Freires I.A., De Alencar S.M., Rosalen P.L. (2016). A Pharmacological Perspective on the Use of Brazilian Red Propolis and Its Isolated Compounds against Human Diseases. Eur. J. Med. Chem..

[355] Bhargava P., Grover A., Nigam N., Kaul A., Doi M., Ishida Y., Kakuta H., Kaul S., Terao K., Wadhwa R. (2018). Anticancer Activity of the Supercritical Extract of Brazilian Green Propolis and Its Active Component, artepillinï¿½C: Bioinformatics and Experimental Analyses of Its Mechanisms of Action. Int. J. Oncol..

[356] Turktekin M., Konac E., Onen H.I., Alp E., Yilmaz A., Menevse S. (2011). Evaluation of the Effects of the Flavonoid Apigenin on Apoptotic Pathway Gene Expression on the Colon Cancer Cell Line (HT29). J. Med. Food.

[357] Li F., Awale S., Tezuka Y., Kadota S. (2008). Cytotoxic Constituents from Brazilian Red Propolis and Their Structure–Activity Relationship. Bioorg. Med. Chem..

[358] Vukovic N.L., Obradovic A.D., Vukic M.D., Jovanovic D., Djurdjevic P.M. (2018). Cytotoxic, Proapoptotic and Antioxidative Potential of Flavonoids Isolated from Propolis against Colon (HCT-116) and Breast (MDA-MB-231) Cancer Cell Lines. Food Res. Int..

[359] Chen J., Sun L. (2012). Formononetin-Induced Apoptosis by Activation of Ras/P38 Mitogen-Activated Protein Kinase in Estrogen Receptor-Positive Human Breast Cancer Cells. Horm. Metab. Res..

[360] Carvalho A.A., Finger D., Machado C.S., Schmidt E.M., Costa P.M.D., Alves A.P.N.N., Morais T.M.F., Queiroz M.G.R.D., Quináia S.P., Rosa M.R.D. (2011). In Vivo Antitumoural Activity and Composition of an Oil Extract of Brazilian Propolis. Food Chem..

[361] Inoue K., Saito M., Kanai T., Kawata T., Shigematsu N., Uno T., Isobe K., Liu C.-H., Ito H. (2008). Anti-Tumor Effects of Water-Soluble Propolis on a Mouse Sarcoma Cell Line In Vivo and In Vitro. Am. J. Chin. Med..

[362] Gal A.F., Stan L., Tăbăran F., Rugină D., Cătoi A.F., Andrei S. (2020). Chemopreventive Effects of Propolis in the MNU-Induced Rat Mammary Tumor Model. Oxid. Med. Cell. Longev..

[363] Ghaderi A., Stice E., Andersson G., Enö Persson J., Allzén E. (2020). A Randomized Controlled Trial of the Effectiveness of Virtually Delivered Body Project (vBP) Groups to Prevent Eating Disorders. J. Consult. Clin. Psychol..

[364] Lee Y.-T., Don M.-J., Hung P.-S., Shen Y.-C., Lo Y.-S., Chang K.-W., Chen C.-F., Ho L.-K. (2005). Cytotoxicity of Phenolic Acid Phenethyl Esters on Oral Cancer Cells. Cancer Lett..

[365] Lavinas F.C., Macedo E.H.B.C., Sá G.B.L., Amaral A.C.F., Silva J.R.A., Azevedo M.M.B., Vieira B.A., Domingos T.F.S., Vermelho A.B., Carneiro C.S. (2019). Brazilian Stingless Bee Propolis and Geopropolis: Promising Sources of Biologically Active Compounds. Rev. Bras. Farm..

[366] Pobiega K., Kraśniewska K., Gniewosz M. (2019). Application of Propolis in Antimicrobial and Antioxidative Protection of Food Quality–A Review. Trends Food Sci. Technol..

[367] Ueda T., Inden M., Shirai K., Sekine S., Masaki Y., Kurita H., Ichihara K., Inuzuka T., Hozumi I. (2017). The Effects of Brazilian Green Propolis That Contains Flavonols against Mutant Copper-Zinc Superoxide Dismutase-Mediated Toxicity. Sci. Rep..

[368] Alghutaimel H., Matoug-Elwerfelli M., Alhaji M., Albawardi F., Nagendrababu V., Dummer P.M.H. (2024). Propolis Use in Dentistry: A Narrative Review of Its Preventive and Therapeutic Applications. Int. Dent. J..

[369] Shinmei Y., Yano H., Kagawa Y., Izawa K., Akagi M., Inoue T., Kamei C. (2009). Effect of Brazilian Propolis on Sneezing and Nasal Rubbing in Experimental Allergic Rhinitis of Mice. Immunopharmacol. Immunotoxicol..

[370] De Barros M.P., Sousa J.P.B., Bastos J.K., De Andrade S.F. (2007). Effect of Brazilian Green Propolis on Experimental Gastric Ulcers in Rats. J. Ethnopharmacol..

[371] Barros M.P.D., Lemos M., Maistro E.L., Leite M.F., Sousa J.P.B., Bastos J.K., Andrade S.F.D. (2008). Evaluation of Antiulcer Activity of the Main Phenolic Acids Found in Brazilian Green Propolis. J. Ethnopharmacol..

[372] Boeing T., Mejia J.A., Ccana-Ccapatinta G.V., Mariott M., de Souza P., Mariano L.N., Oliveira G.R., da Rocha I.M., da Costa G.A., de Andrade S.F. (2021). The Gastroprotective Effect of Red Propolis Extract from Northeastern Brazil and the Role of Its Isolated Compounds. J. Ethnopharmacol..

[373] Silva-Carvalho R., Baltazar F., Almeida-Aguiar C. (2015). Propolis: A Complex Natural Product with a Plethora of Biological Activities That Can Be Explored for Drug Development. Evid.-Based Complement. Altern. Med..

[374] Spinelli S., Conte A., Lecce L., Incoronato A.L., Del Nobile M.A. (2015). Microencapsulated Propolis to Enhance the Antioxidant Properties of Fresh Fish Burgers. J. Food Process Eng..

